# Comparative analysis of the AIB1 interactome in breast cancer reveals MTA2 as a repressive partner which silences E-Cadherin to promote EMT and associates with a pro-metastatic phenotype

**DOI:** 10.1038/s41388-020-01606-3

**Published:** 2021-01-08

**Authors:** Damir Varešlija, Elspeth Ward, Siobhan P. Purcell, Nicola S. Cosgrove, Sinéad Cocchiglia, Philip J. O’Halloran, Sara Charmsaz, Fiona T. Bane, Francesca M. Brett, Michael Farrell, Jane Cryan, Alan Beausang, Lance Hudson, Arran K. Turnbul, J. Michael Dixon, Arnold D. K. Hill, Nolan Priedigkeit, Steffi Oesterreich, Adrian V. Lee, Andrew H. Sims, Aisling M. Redmond, Jason S. Carroll, Leonie S. Young

**Affiliations:** 1 Endocrine Oncology Research Group, Department of Surgery, Royal College of Surgeons in Ireland, Dublin, Ireland; 2 Department of Neurosurgery, National Neurosurgical Center, Beaumont Hospital, Dublin, Ireland; 3 Department of Neuropathology, National Neurosurgical Center, Beaumont Hospital, Dublin, Ireland; 4 Breast Cancer Now Research Laboratories, Edinburgh, EH4 2XU UK; 5 Department of Pharmacology and Chemical Biology, University of Pittsburgh Cancer Institute, University of Pittsburgh, Pittsburgh, PA USA; 6 Department of Women’s Cancer Research Center, Magee-Women’s Research Institute, University of Pittsburgh Cancer Institute, University of Pittsburgh, Pittsburgh, PA USA; 7 Department of Human Genetics, University of Pittsburgh Cancer Institute, University of Pittsburgh, Pittsburgh, PA USA; 8 Applied Bioinformatics of Cancer Group, University of Edinburgh Cancer Research UK Centre, MRC Institute of Genetics & Molecular Medicine, Western General Hospital, Edinburgh, UK; 9 Cancer Research UK Cambridge Institute, University of Cambridge, Li Ka Shing Centre, Robinson Way, Cambridge, CB2 0RE UK

**Keywords:** Breast cancer, Metastasis

## Abstract

Steroid regulated cancer cells use nuclear receptors and associated regulatory proteins to orchestrate transcriptional networks to drive disease progression. In primary breast cancer, the coactivator AIB1 promotes estrogen receptor (ER) transcriptional activity to enhance cell proliferation. The function of the coactivator in ER^+^ metastasis however is not established. Here we describe AIB1 as a survival factor, regulator of pro-metastatic transcriptional pathways and a promising actionable target. Genomic alterations and functional expression of AIB1 associated with reduced disease-free survival in patients and enhanced metastatic capacity in novel CDX and PDX ex-vivo models of ER^+^ metastatic disease. Comparative analysis of the AIB1 interactome with complementary RNAseq characterized AIB1 as a transcriptional repressor. Specifically, we report that AIB1 interacts with MTA2 to form a repressive complex, inhibiting CDH1 (encoding E-cadherin) to promote EMT and drive progression. We further report that pharmacological and genetic inhibition of AIB1 demonstrates significant anti-proliferative activity in patient-derived models establishing AIB1 as a viable strategy to target endocrine resistant metastasis. This work defines a novel role for AIB1 in the regulation of EMT through transcriptional repression in advanced cancer cells with a considerable implication for prognosis and therapeutic interventions.

## Introduction

Breast cancer metastases arising from luminal tumors have been poorly understood despite representing the most common molecular subtype. The shift to a metastatic phenotype encompasses dynamic and progressive alterations in key (epi)-genetic and transcriptomic processes occurring in the face of dysregulated steroid hormone signaling [1]. Primary luminal breast tumors typically express estrogen receptor (ER) and are reliant on nuclear receptors and transcriptional coregulators to operate key nodes required for the crosstalk between endocrine and growth factor signaling [1]. Given that the pro-proliferative transcriptional response is self-sustaining in the absence of steroids, understanding the regulatory machinery driving the transition to endocrine therapy resistance and a switch to metastatic tumor formation becomes essential.

Endocrine therapy resistant tumor cells can frequently undergo adaptive transcriptional reprogramming resulting in the diminished dependence on steroid hormone signaling [2–4]. Other growth and survival mechanisms may therefore emerge in many cases even if ER remains expressed, but where its activity is bypassed. Our previous work identified steroid receptor coactivator, AIB1 (SRC3, NCOA3), as a key determinant of the adaptive response in endocrine resistance [2, 5]. AIB1 is frequently amplified and over-expressed in a number of epithelial tumors [6]. Altered expression of AIB1 in breast cancer contributes to tumor initiation and progression [5, 7, 8] and has been specifically associated with the early survival of cells that have acquired endocrine resistance [2]. In addition, previous work has suggested that the advanced endocrine resistant metastatic phenotype appears to be less dependent on ER and its transcriptional program [2, 5]. These prior observations support the idea that in endocrine resistant tumors, AIB1 may not act exclusively via ER and its association with other factors likely explains its continued function in metastasis.

The pro-metastatic AIB1-interacting network is poorly characterized and the mechanism that bypasses ER activity and employs AIB1 in the metastatic phenotype in endocrine resistant disease remains unknown. To shed light on these issues, we establish that AIB1 acts a key survival factor in advanced breast cancer models where it activates transcriptional programs associated with endocrine resistance. We use rapid immunoprecipitation mass spectrometry of endogenous proteins (RIME) to define the AIB1 interactome mediating a pro-metastatic phenotype under endocrine resistant conditions. We demonstrate that AIB1 can interacts with MTA2 to form a repressive complex, inhibiting CDH1 (encoding E-cadherin) to promote EMT and drive progression in ER-positive luminal tumors. Consequentially, AIB1 may represent a promising therapeutic opportunity in the metastatic phase of endocrine resistant disease. These findings emphasize the importance of AIB1 in ER+ metastatic breast cancer pathology and establish AIB1-dependent functions as a key molecular determinant of metastatic competence.

## Results

### AIB1 is enriched in endocrine resistant and metastatic tumors where it associates with poor outcome

**Fig. 1:**
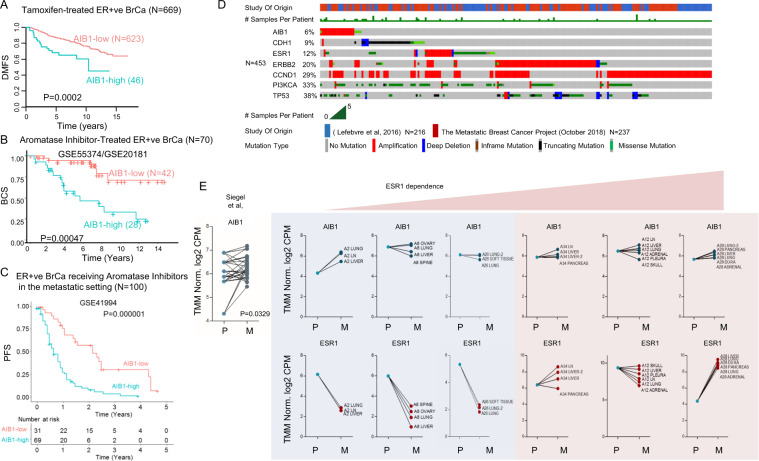
AIB1 predicts outcome in endocrine resistant breast cancer. **A** Ranked AIB1 target gene set expression in 669 primary breast tumors from ER positive Tamoxifen-treated patients. Kaplan–Meier analysis of distant metastatic free survival (DMFS) according to mRNA expression of AIB1 in ER positive 4-OHT-treated patients (*n* = 669). Data is from four published Affymetrix microarray datasets (GSE6532, GSE9195, GSE17705, GSE12093). **B** Ranked AIB1 expression in primary breast tumors from ER positive AI-treated patients. AIB1 mRNA expression associated significantly with worse breast cancer specific survival (BCS) (*p* = 0.0004, *n* = 70, HR 4.7). Data is from published Affymetrix microarray datasets (GSE55374/GSE20181). For both colors are log2 mean-centered values, blue=high, orange=low. All data sets were summarized with Ensembl alternative CDF and normalized with Robust Multi-array Average (RMA), before integration using ComBat to remove dataset-specific bias. **C** Ranked AIB1 expression in metastatic breast tumors from ER+ AI-treated patients. Kaplan-Meier displays high AIB1 expression associating with worse progression-free survival (*p* = 0.000001, *n* = 100) in metastatic ER+ AI-treated patients. Data is from published Affymetrix microarray dataset GSE41994. Colors are log2 mean-centered values, blue=high, orange=low. All data sets were summarized with Ensembl alternative CDF and normalized with Robust Multi-array Average (RMA), before integration using ComBat to remove dataset-specific bias. **D** cBioPortal generated oncoprint map displaying most common amplifications/mutations detected in DNA-sequenced metastatic breast tumors from the Metastatic Breast Cancer Project and Lefebre et al cohort (*n* = 453). Oncoprint map is cropped to highlight first 100 tumors that capture AIB1 aberrations in relation to other frequently altered genes (Full annotation can be found in Supplement Tables). **E** Paired ladder plot of AIB1 and ESR1 mRNA expression in patient-matched primary (P) and metastatic (M) cases (*n* = 6 patients; *n* = 32 tumors; Siegel et al.). Light green dots represent primary tumor expression values and dark green dots represent metastatic tumor expression values (log2norm CPM). *P* value obtained via two-sided Wilcoxon signed-rank test.

Associations between aberrant expression of AIB1 and poor prognosis in ER^+^ breast tumors have been previously reported by our group and others [5, 9, 10]. Consistent with these studies, here, in extended cohorts of endocrine therapy treated populations, we observed worse distant metastasis free survival in tamoxifen treated patients and reduced breast cancer specific survival in AI treated patients harboring high AIB1 mRNA expression (*P* = 0.0002, *N* = 669; *P* = 0.0004, *N* = 70, respectively) (Figs. 1A, B; S1A, B). Associations of elevated AIB1 mRNA with worse overall survival were further validated in ER+ tumors from the METABRIC cohort (*P* = 0.0259; *N* = 1825) (Fig. S1C, Supplementary Table S1a). Given the lack of knowledge regarding the contribution of AIB1 in ER^+^ metastasis, we interrogated an additional independent cohort of ER^+^ patients receiving endocrine treatment in the metastatic setting. Here, high AIB1 expression associated with worse progression-free survival in the advanced patient population (*P* = 0.000001, HR = 3.6, *n* = 100) (Fig. 1C).

In addition we investigated the frequency of AIB1 genomic alterations in the publically available Metastatic Breast Project and the Lefebvre et al. metastatic cohorts (*N* = 453) [11]. AIB1 amplifications/mutations were detected in 6% of tumors and are displayed alongside genes found frequently mutated in metastatic patients, including ESR1, PI3KC and ERBB2 [12–14] (Fig. 1D, Table S1b). Of interest, the majority of AIB1 genomic alterations were found to be independent of ESR1, ERBB2 or PI3KC mutations (co-occurrence tendency with AIB1: *P* = 0.536, *P* = 0.543, and *P* = 0.385 respectively) (Fig. 1D, Table S2).

This lack of coassociation between ESR1 and AIB1-driven metastatic tumors was further substantiated in the analysis of Siegel et al. RNA-seq of ER^+^ primary breast tumors and matched metastasis from multiple organs (*N* = 6 patients; *N* = 32 tumors) [15]. Here, while ESR1 expression is not routinely maintained, the metastatic tumors (including lung, liver, lymph nodes, and adrenal) consistently sustain or harbor increases in AIB1 mRNA expression in comparison with the primary tumors (*P* = 0.0329) (Fig. 1E). To corroborate these observations we analyzed RNA-seq data in our cohort of longitudinal ER^+^ patient–matched primaries and metastatic tumors (bone and brain; *n* = 34), where we observe a decrease in ESR1 expression (*P* = 0.0371) and its target gene GREB1 (*P* = 0.0137) on metastasis, with no loss detected in AIB1 mRNA expression (*P* = 0.097) (Fig. S1D). Moreover, genome-wide analysis of transcriptionally activating H3K4me3 mark in endocrine resistant metastatic tumors by ChIP-seq [16] (*n* = 10) revealed enhanced activity at AIB1 loci in bad outcome versus good outcome metastatic tumors (Fig. S1E). Collectively, these findings demonstrate a clear association between high AIB1 mRNA expression and poor outcome in endocrine therapy treated ER^+^ tumors that carries into the metastatic setting.

### Inhibition of AIB1 function demonstrates significant anti-tumor activity in endocrine resistance

**Fig. 2:**
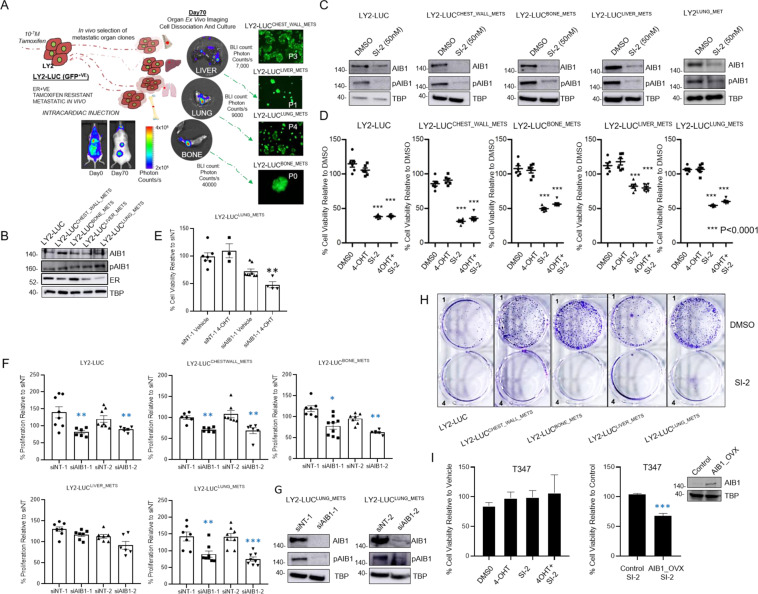
AIB1 can be targeted in models of metastatic disease. **A** Schematic representation of the in vivo isolation of the metastatic endocrine resistant cells. LY2-Luc cells were disseminated via intracardiac injection into NOD-SCID mice. At the end of experiment the metastatic cells were confirmed by IVIS bioluminescence imaging (BLI) in vivo and ex vivo. Metastatic colonising cells from chest-wall, liver, lung, and bone were isolated, briefly cultured and injected 3 more times. These cells were once again isolated and metastatic variants were established in culture. Representative images with indicated BLI readings and scale are displayed for in vivo and ex vivo imaging alongside 20x FITC (fluorescein isothiocyanate) microscopy images of early cultured GFP + ve cells. **B** Western blotting of AIB1, pAIB1, ER, and TBP protein expression in the nuclear lysates of various endocrine resistant metastatic variants under estrogen deprived conditions. **C** AIB1 and pAIB1 western blots 48 h post treatment with 50 nM of AIB1 inhibitor SI-2. **D** Cells were treated for 96 h with vehicle (DMSO), 4-OHT (10-7 M) or SI-2 (50 nM) under estrogen deprived conditions. **E** Cells were transfected with either siNT-1 or siAIB1-1 and cell viability assay was carried out in the presence of either vehicle or 4-OHT (10-7 M). Cell viability was measured using an MTS assay and graph display % cell viability relative to vehicle. **F** Cells were transfected with either siNT-1 and siNT-2 or siAIB1-1 and siAIB1-2. Cell proliferation was measured after 5 days. Graphs display % proliferation relative to siNT. **G** AIB1 and pAIB1 western blots 48 h post transfection with siNT-1, siNT-2, siAIB1-1, or siAIB1-2. **H** Colony forming assay was carried out with cells seeded under estrogen depleted conditions and treated with DMSO or 50 nM SI-2 every 3 days for a total of 14 days. Representative images of the cells stained with crystal violet at day 14. **I** T347 primary culture cell line was treated for 96 h with vehicle (DMSO), 4-OHT (10-7 M) or SI-2 (50 nM). T347 cells were transfected with either empty pcDNA3.1 plasmid vector (Control) or pcDNA3.1 plasmid containing full-length AIB1 (AIB1_OVX) which were both treated with DMSO and SI-2 (50 nM) for 96 h and cell viability measured. Graph displays % cell viability relative to Vehicle control. Western blot analysis of AIB1 and TBP. All experiments are representative of three biologically independent replicates. Western blot gel images are cropped at the band of interest for clarity. Two-sided t-tests were used to calculate *P* values (***P < 0.0001).

To investigate the contribution of AIB1 in ER^+^ metastasis we utilized endocrine resistant LY2 cells that form metastases following both mammary fat pad implantation [3] and intracardiac injection. LY2 cells are resistant to both tamoxifen and insensitive to the selective ER degrader, fulvestrant, in vitro and ex vivo (Fig. S2A, B). Through multiple rounds of intracardiac injections, we established metastatic sub-lines of LY2 cells that colonized the chest-wall, liver, lung, and bone (Fig. 2A). Alongside their parental LY2, all sublines expressed AIB1 and pAIB1^Thr24^ protein with varying levels of expression of ER (Fig. 2B). Previous studies have reported on a new class of potent small molecule inhibitor against AIB1, SI-2 [17]. SI-2 has been shown to selectively target protein levels of AIB1 and its transcriptional activity with effective activity in vivo and minimal toxicity observed [17, 18]. Using LY2-LUC cells and each of the corresponding metastatic sub-lines we were able to demonstrate that treatment with an AIB1 inhibitor, SI-2, can reduce expression of both total AIB1 and pAIB1^Thr24^ (Fig. 2C). Indeed, short-term (96 h) treatment of endocrine resistant metastatic sub-lines with SI-2 demonstrated a significant decrease in cell viability compared to vehicle or endocrine treatment alone (Fig. 2D). Moreover, SI-2 treatment appears more efficacious compared to both fulvestrant and palbociclib in LY2-LUC and LY2-LUC^Lung_Met^, while performing comparably in LY2-LUC^Bone_Met^ (Supplementary Fig. S2C). Given that AIB1 is a mediator of endocrine treatment resistance we find that inhibition of AIB1 by knockdown in resistant metastatic cells LY2-LUC^LUNG_METS^ is sufficient to induce a re-sensitization of resistant cells to 4-OHT (Fig. 2E).

In addition, using two independent siRNAs directed against AIB1 we observe a significant reduction in cell proliferation in most of the endocrine resistant metastatic sub-lines (Fig. 2F) with a noted downregulation in protein levels of AIB1 and pAIB1^Thr24^ (Fig. 2G). The impact of AIB1 inhibition on cell growth of metastatic sub-lines was even more pronounced over a 14 day treatment as evidenced by colony forming assay (Fig. 2H). Using LY2-LUC model we demonstrate that a knockdown of AIB1 can diminish the effectiveness of SI-2 inhibition suggesting specific action against AIB1 (Supplementary Fig. 2D). This is further substantiated by the lack of response to SI-2 observed in the ER^+ve^ endocrine resistant T347 cell line model (AIB1 low protein expression), which can be induced with AIB1 overexpression (Fig. 2I). Taken together, these observations reinforce the continued involvement of AIB1 in endocrine resistant metastatic setting.

**Fig. 3:**
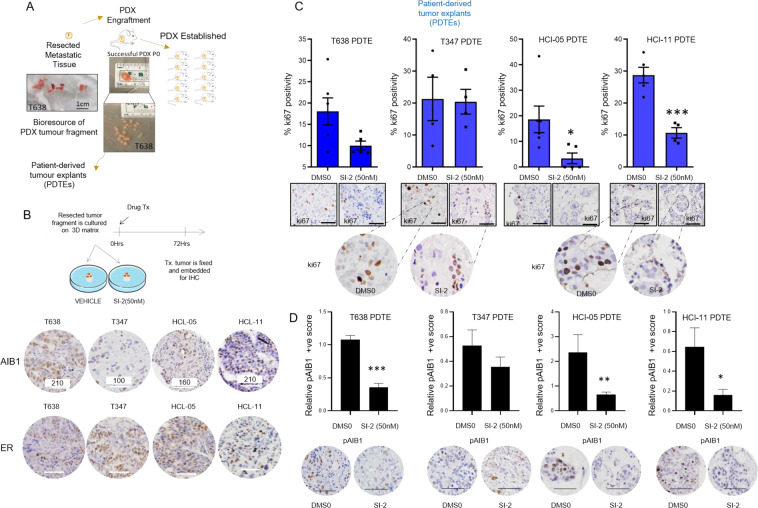
Inhibition of AIB1 demonstrates significant anti-tumor activity in endocrine resistant patient-derived metastatic samples. **A** Schematic indicates establishment protocol for patient-derived xenografts (PDXs) and subsequent patient-derived tumor explants (PDTEs). **B** Schematic of the ex vivo PDTE experiment set up. Immunohistochemistry (IHC) protein analysis of AIB1 and ER in PDX models established from metastatic endocrine resistant patient samples. Various PDXs were confirmed to have variable levels of AIB1 protein expression. H score is displayed and was quantified from three biologically independent samples. At least 500 cells were assessed in each case. **C** PDTEs were treated with DMSO or 50 nM SI-2 and processed as described. Bar chart displays ki67% determined from manual counts of ki67 positive cells over total number of cells. Representative images of IHC analyses of Ki67 tumors treated for 72 h with indicated treatments. Zoomed in images of ki67+ve representative areas of worst responder PDTE T347 and best responder HCI-11. All scale bars represent 50 μm. Error bars represent mean ± s.e.m. (*n* = 4–6 images per group). **D** Bar chart displays % pAIB1^Thr24^(pAIB1) positivity relative to DMSO determined by Aperio ImageScope positive pixel algorithm. Representative IHC images of pAIB1 in DMSO and SI-2 treated samples. Two-sided *t* tests were used to calculate *P* values. All scale bars, 50 μm.

Next, we evaluated the impact of targeting AIB1 in several ER^+^ metastatic patient-derived xenografts (PDXs) [19–21] that model clinically relevant endocrine resistant tumors (Table S3). PDXs were engrafted from metastatic tissue and used to further establish patient-derived tumor explants (PDTEs) (Fig. 3A). Tumor tissue from five PDTEs with varying AIB1 protein positivity scores was treated with 50 nM of AIB1 inhibitor SI-2 for 72 h and the proliferation index measured (Fig. 3B). SI-2 treatment inhibited tumor proliferation and pAIB1^Thr24^ protein expression in high pAIB1 models including T638 PDTE (ki67% inhibition 44%; *P* = 0.055), HCI-05 PDTE (ki67% inhibition 82%; *P* = 0.032) and HCI-11 PDTE (ki67% inhibition 63%; *P* < 0.001), but not the low pAIB1-expressing T347 and T328 PDTE model (Fig. 3C and D, Supplementary Fig. 3A). By analyzing pAIB1^Thr24^ levels for T638, T347, HCI-11, and HCI-05 in DMSO versus SI-2, we observe a significant reduction in the levels of pAIB1 in the responder tumor explants (Fig. 3D). In the case of tumor explant T638 where we had sufficient tissue sections, we also profiled AIB1 and pER^SER118^ and we observe a significant reduction in total AIB1 levels but not pER^SER118^ (Supplementary Fig. 3B).

Similarly, to the LY2-LUC^LUNG_METS^, a targeted approach utilizing SI-2 in the PDTE T638 is sufficient to induce re-sensitization of resistant tumor to antiestrogens (Supplementary Fig. 3C). Together, these data underpin the significance of AIB1 in endocrine resistant ER^+^ metastatic breast cancer pathology and substantiate the potential of AIB1 as a therapeutic target in advanced disease.

### AIB1-dependent transcriptome and interactome as potentiators of the pro-metastatic phenotype

**Fig. 4:**
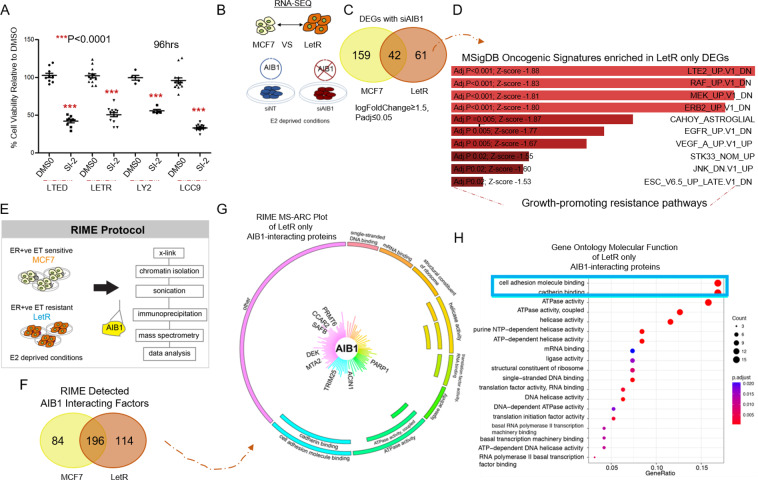
AIB1 transcriptome and interactome in the endocrine resistant cells acts as a regulator of pro-metastases pathways. **A** Scatter plot graph displays % cell viability after 96 h of multiple endocrine resistant cell line models (under estrogen deprived conditions) in the presence of vehicle (DMSO) or AIB1 inhibitor SI-2 (50 nM). All results are mean ± S.E.M., *n* = 3 and two-sided *t* tests were used to calculate *P* values (***P < 0.0001). **B** Schematic of the RNA-seq experimental design. RNA-seq was carried out in biological triplicate in MCF7 and LetR cells comparing NT (Scramble siRNA) cells versus cells depleted of AIB1 using a SMARTpool ON-TARGETplus siRNA. **C** Venn diagram displays the number of differentially expressed genes (DEGs) identified with AIB1 knockdown in MCF7 versus LetR cells (logFoldChange ± 1.5, Padj ≤ 0.05). **D** Graph displays MsigDB Oncogenic signature gene pathways enriched in AIB1-dependent DEGs specific to LetR cells. Also displayed are *P* values and Z scores for reach significant pathway identified. **E** Schematic representation of the experimental design for Rapid Immunoprecipitation of Endogenous (RIME) AIB1. AIB1 RIME was conducted in MCF-7 and LetR cells under estrogen deprivation conditions (*n* = 3 biological replicates). Nonspecific interactions (identified from multiple IgG control replicates) are removed. ET = endocrine therapy. (F) Venn diagram displays AIB1-interacting proteins identified in MCF7 and LetR. **G** MS-ARC plot shows AIB1-associated proteins specific to LetR cells. The AIB1-associated proteins are clustered according to molecular function, and the length of the line represents the Mascot score. Proteins of interest are highlighted with enlarged bold text. AIB1 is displayed in the MS-ARC plot as the immunoprecipitated protein of interest. (H) Plot of the Gene Ontology (GO) molecular function enrichment test for the LetR only AIB1 associated proteins (hypergeometric test, P-adj ≤ 0.05).

Having observed the transcriptomic/genomic divergence between AIB1-driven tumors and its known partnering factors, it was important to define the mechanism behind pro-metastatic phenotype potentiated by AIB1. To do this we utilised the endocrine resistant cell models that express AIB1 and respond to pharmacological AIB1 inhibition under estrogen-deprived conditions (Figs. 4A, S4A, B). Given that we saw the most significant clinical association of high AIB1 expression and metastatic progression in AI-treated patient models, we decided to further investigate AIB1 in the AI-resistant, LetR cells. LetR cells were established from the parental MCF7 cells with a forced overexpression of CYP19 and long-term treatment with letrozole [2, 5].

In transient knockdowns in estrogen deprived LetR cells, loss of AIB1 inhibited classic mechanism of epithelial tumorigenicity including cell viability, mammosphere formation and putative stemness (Fig. S4C). To elucidate the aberrant global transcriptional program regulated by AIB1, we undertook RNA-seq analysis in endocrine resistant LetR cells and endocrine sensitive MCF7 cells, in the presence and absence of AIB1 under estrogen deprivation (Figs. 4B; S4D, E; Table S4, 5). Sixty-one AIB1 differentially regulated genes were identified as unique to endocrine resistant tumor cells (logfc ≥ 1.5; Padj ≤ 0.05) (Fig. 4C, Table S6). Of interest this gene set does not overlap with the previously identified AIB1 coactivated subset of ESR1 dysregulated genes [2]. MSigDB Oncogenic signature analysis of the 61 AIB1-regulated genes revealed enrichment in growth-promoting pathways previously associated with endocrine resistance including LTE2, RAF, ERBB2, and MEK (Fig. 4D; Table S7).

To better understand the mechanism behind the endocrine resistant-specific gene programs regulated by AIB1 it was important to define the AIB1 interactome in this setting. We employed RIME AIB1 protein [22] in endocrine resistant LetR cells and endocrine sensitive MCF7 cells, both under estrogen deprived conditions (Fig. 4E). We solely considered AIB1-interacting proteins that were common in three out of three independent biological replicates and not in IgG controls. This approach yielded 280 AIB1-interacting proteins in MCF7s and 310 AIB1-interacting proteins in LetR cells (Fig. 4F; Table S8). ER was not pulled out as an interacting partner of AIB1 in the absence of estrogen, as has been described previously [22, 23]. The estrogen-dependent AIB1-ER interaction was verified using a standard Co-IP experiment in MCF7s (Fig. S5A). One hundred and fourteen AIB1 interacting proteins were found to be unique to LetR cells (Fig. 4F, G; Table S9), including known pro-tumorigenic and pro-metastatic proteins, such as MTA2, DEK, TRIM25, CCAR2, RAC1, RUVB1, ACIN1.

To better understand the potentially divergent molecular processes potentiated and mediated by AIB1 and to prioritize essential genes, we performed molecular function pathway enrichment analysis of the interacting proteins unique to LetR. Cell adhesion and cadherin binding, key constituents of epithelial to mesenchymal transition (EMT), were amongst the top-ranked AIB1 interacting proteins (Fig. 4H, Table S10). Consistent with this observation, KEGG pathway analysis of differentially expressed genes (DEGs) from our RNAseq studies revealed that endocrine resistant specific DEGs were also enriched for a number of metastases promoting EMT pathways (e.g., ECM-receptor interaction, Rap1 signaling, axon guidance, focal adhesion and cell adhesion molecules) (Fig. S5B, C; Table S11, S12). Further clinical relevance of the endocrine resistant AIB1 cell adhesion/cadherin binding interactome was examined in the same cohort of metastatic ER^+^ endocrine-treated breast cancer patients. Comprehensive progression-free survival analysis for the 16 top-ranked AIB-1 interacting proteins demonstrated that elevated levels of 11/16 genes associated with worse PFS (Fig. S5D; Table S13). These findings suggest a role for AIB1 in mediating a pro-metastatic EMT program in ER^+^ breast cancer.

### AIB1 interactome as a regulator of pro-EMT phenotype

**Fig. 5:**
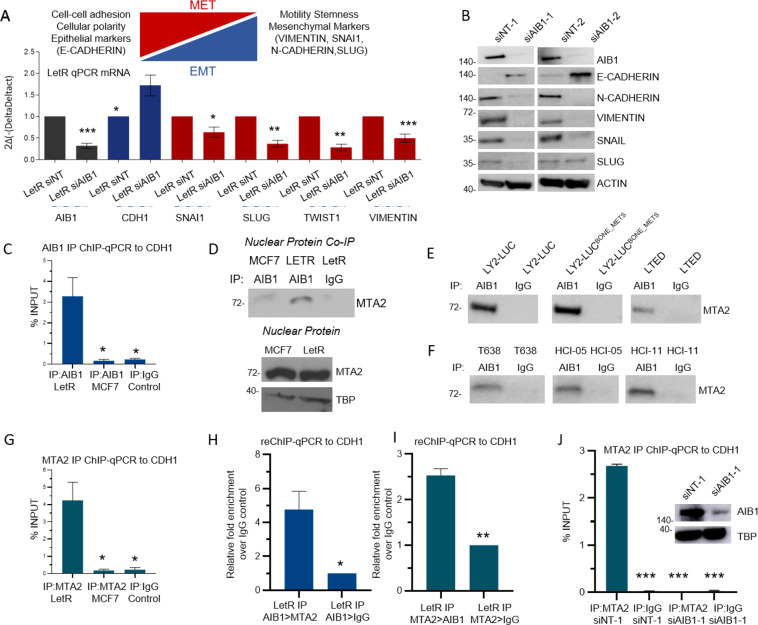
AIB1 induces and regulates EMT and CDH1. **A** qRT-PCR analysis of selected EMT markers gene expression in the LetR cells transiently transfected with either NT or AIB1 siRNA. **B** Western blot analysis of AIB1, B-ACTIN, and EMT markers N-cadherin, E-cadherin, Vimentin, Snail and Slug. LetR cells were transfected with either siNT-1, siNT-2, siAIB1-1, or siAIB1-2 and whole cell lysate harvested. In total, 30 µg of protein was loaded per gel. **C** AIB1 Chromatin Immunoprecipitation (ChIP) assay in MCF7 and LetR cells showing the differential recruitment of IP AIB1 to the CDH1 promoter. CHIP-grade Rabbit IgG antibody was used as a control and data are presented as % INPUT. **D** Co-Immunoprecipitation (CO-IP) of AIB1 followed by western immunoblotting to detect the MTA2 interaction in nuclear extracts of LetR and MCF7 cells CHIP-grade Rabbit IgG used as a control. Western blotting of MTA2 protein expression in the nuclear lysates. **E**, **F** CO-IP of AIB1 in nuclear extracts of endocrine resistant cell lines and PDX tumors followed by immunoblotting for MTA2. CHIP-grade Rabbit IgG used as a control. **G** MTA2 IP ChIP in MCF7 and LetR cells showing the differential recruitment of MTA2 on the CDH1 promoter. Data are presented as % INPUT. **H** CHIP-re-ChIP in LetR cells displaying relative fold enrichment over control with AIB1-IP > MTA2-IP versus AIB1-IP > IgG-IP. **I** CHIP-re-ChIP in LetR cells displaying relative fold enrichment over control with MTA2-IP > AIB1-IP versus MTA2-IP > IgG-IP. **J** MTA2 IP ChIP showing the recruitment of MTA2 on the CDH1 promoter in siNT-1 versus si-AIB1 LetR cels. Data are presented as % INPUT. All experiments carried out under steroid starved conditions with the exception of PDX tumors in (**F**). ChIP-grade Rabbit IgG used as a control in ChIP, re-ChIP and Co-IP experiments. Western blot gel images are cropped at the band of interest for clarity. No other bands were detected in Co-IP gels. All experiments are representative of three biologically independent replicates. In the case of PDX tumors protein was pooled. Two-sided t-tests were used to calculate *P* values.

In the context of endocrine resistance, little is known about the function of AIB1 in regulating EMT and pro-metastatic phenotype. Initiation and establishment of metastasis is dependent on EMT associated transcription factors (TFs) and key EMT factors including the prototypic cell adhesion molecule, e-cadherin (encoded by *CDH1*) [24, 25]. Here, we find that AIB1 differentially regulates gene expression of a number of EMT drivers identified above with suppression of *CDH1* and up regulation key EMT TFs including SLUG and TWIST (Fig. 5A). Utilizing two independent siRNA against AIB1, we confirm these alterations at protein level with a reduction in levels of N-cadherin, Vimentin, Snail and an increase in E-cadherin (Fig. 5B).

Genome-wide analysis of the transcriptionally activating H3K4me3 mark in endocrine resistant metastatic tumors, revealed altered binding profiles in poor outcome patient tumors for EMT marker genes profiled in 5 A and genes encoding the top-ranked AIB1 interacting cell adhesion and cadherin binding proteins (Fig. S6A, B). These data prompted a further investigation into transcriptional regulation of pro-EMT pathways in endocrine resistance and in particular unexpected *CDH1* repression by the coactivator AIB1.

AIB1 IP ChIP-qPCR revealed that AIB1 preferentially bound to the promoter region of CDH1 in LetR cells when compared to MCF7 cells, supporting the enriched cell adhesion/cadherin binding phenotype identified in AIB1-interactome in LetR only, but not MCF7 cells (Fig. 5C). Interestingly, ER itself was previously shown to bind CDH1 and repress E-cadherin expression in estrogen stimulated MCF7 cells [26]. This was confirmed by identifying ESR1 as the most frequent breast cancer cell-related factor binding to the CDH1 promoter sequence using the DB cistrome toolkit [27] (Table S14). However, ER IP followed by ChIP-qPCR demonstrated no ER binding to CDH1 promoter in the endocrine resistant LetR under the same conditions. In contrast and in agreement with previously published studies, estrogen stimulated ER binding to CDH1 in endocrine sensitive MCF7 cells was evident (Fig. S6 C, D).

Although previously reported for members of the nuclear coactivator family SRC-1 and SRC-2, transcriptional repression potentiated by AIB1 has yet to be fully elucidated [19, 28–32]. Accumulating data indicates that AIB1 typically acts as a primary recruiter of additional coregulators that execute its transcriptional regulation as evidenced in its recruitment of activators, p300/CBP and CARM1 [33]. Here we looked for potential AIB1 interactors detected by RIME that could be executing AIB1-mediated repression of CDH1. A number of chromatin binding proteins with known repressive function, including MTA2 previously identified to repress both ESR1 and CDH1, were identified as AIB1 partners [34, 35]. To further support the endogenous interaction between AIB1 and MTA2, nuclear protein from MCF7 and LetR cells, under estrogen-starved conditions, was evaluated by a standard coimmunoprecipitation (Co-IP) experiment. AIB1 IP, followed by immunoblotting against MTA2 demonstrated that MTA2 was consistently and differentially coimmunoprecipitated with AIB1 in LetR cells (Fig. 5D), thus confirming it as a potential partner. The increased interaction between AIB1/MTA2 in LetR cells was independent of basal MTA2 expression (Fig. 5C). Moreover, AIB1 IP, followed by immunoblotting against MTA2 confirmed AIB1/MTA2 interaction in LY2-LUC, metastatic variant LY2-LUC^Bone_Mets^ and LTED cells (Fig. 5E). This was also true in the case of patient-derived tumors T638, HCI-05 and HCI-11 that responded to AIB1 inhibition with SI-2 (Fig. 5F).

Similar to AIB1, MTA2 IP followed by ChIP-qPCR to the CDH1 gene was found to be significantly enriched over IgG in LetR cells and no binding was detected in MCF7 cells under the same conditions (Fig. 5G). Other identified cofactors with known repressor activity do not bind to the same region in LetR cells, as was the case with TRIM25 and HDAC2 (Fig. S6E). Analysis of publically available AIB1 and MTA2 ChIP-seq data revealed majority DNA binding events were shared with only 3% of the binding peaks differentially expressed between AIB1 and MTA2 and a notable overlap with the AIB1 differentially regulate genes identified in Fig. 4 (Supplementary Fig. 7, Supplementary Table S15). The co-occupancy was corroborated with a ChIP-reChIP in LetR cells utilizing AIB1-MTA2 and MTA2-AIB1 sequential IPs. Re-ChIP-qPCR revealed that AIB1-MTA2 binding to the CDH1 was significantly enriched over AIB1-IgG (Fold Enrichment 4.7 over IgG, *P* = 0.025) in LetR cells (Fig. 5H). Similarly, a reverse MTA2-AIB1 re-ChIP-qPCR demonstrated an enriched binding over MTA2-IgG (Fold Enrichment 2.5 over IgG, *P* = 0.018), comparably less than AIB1-MTA2 (Fig. 5I). Finally, an AIB1 knockdown with siAIB1-1 was sufficient to reduce MTA2 binding to the CDH1 gene promoter region (Fig. 5J).

**Fig. 6:**
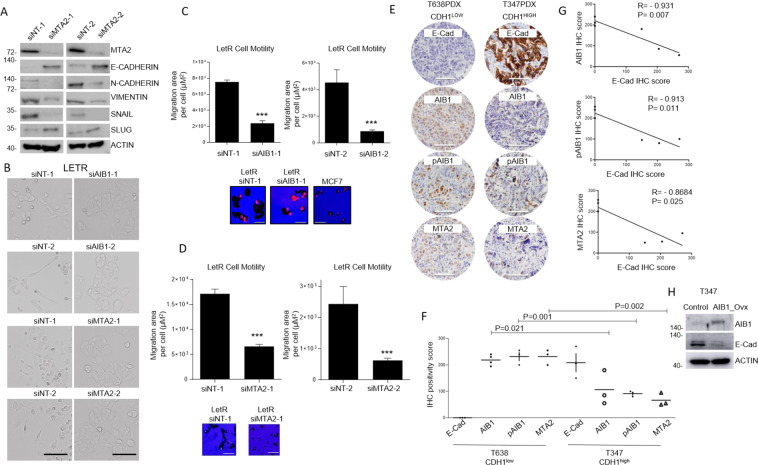
AIB1 interactome as a regulator of pro-EMT phenotype. **A** Western blot analysis of MTA2, B-ACTIN, and EMT markers N-cadherin, E-cadherin, Vimentin, Snail and Slug. LetR cells were transfected with either siNT-1, siNT-2, siMTA2-1, or siMTA2-2 and whole cell lysate harvested. 30µg of protein was loaded per gel. Western blot gel images are cropped at the band of interest for clarity. **B** Light-phase microscopy images of LetR cells cellular characteristics in the presence of two distinct siRNAs against AIB1 and MTA2. Cells were steroid and serum-starved and then subsequently seeded in presence of serum. Representative images of an *n* = 3 biological replicates taken 48 h after seeding (×20 magnification, scale bars represent 100 μm). **C**, **D** LetR cells were depleted of AIB1 or MTA2 using a two independent siRNAs. Cellomics Cell Motility Kit was used to assess individual cell movement in collagen in a 96-well plate after 24 h. Representative images are shown as seen on the microscope after cells were fixed and stained (×20 magnification, scale bars represent 20 μm). All functional experiments were carried out under estrogen deprived conditions. Image of treatment sensitive cell line MCF7 is shown for comparison. Histogram shows mean migratory area per cell (μm.^2^). All results in the figure are mean ± S.E.M., *n* = 3 (biologically independent replicates) and two-sided *t* tests were used to calculate *P* values (*P < 0.05; **P < 0.001, ***P < 0.0001). **E** Representative images of E-Cadherin (E-Cad), AIB1, pAIB1Thr24, and MTA2 protein immunohistochemistry (IHC) in PDX tumor T638- PDX (left panel) and T347-PDX (right panel). All scale bars, 50 μm. **F** Scatter plot of IHC scores (0–300) calculated in T638-PDX and T347-PDX (*n* = 3 mice per PDX model; At least 500 cells were assessed in each case). **G** Correlation plot comparing IHC scores in T638-PDX and T347-PDX, assessing AIB1vCDH1, pAIB1vCDH1, and MTA2vCDH1. **H** Western blot analysis of E-cadherin (E-Cad) protein expression (from whole cell lysate) in the T347x primary culture cells transfected with either empty pcDNA3.1 plasmid vector (Control) or pcDNA3.1 plasmid containing full-length AIB1 (AIB1_OVX). Gel images display AIB1, E-Cadherin (E-Cad) and B-Actin and are representative of three biologically independent replicates.

In light of AIB1 and MTA2 co-operation, we assessed the functional consequence of their expression in the LetR model. Similarly, to AIB1, MTA2 was found to regulate key EMT markers at protein level with an observed reduction in levels of N-cadherin, Vimentin, Snail and an increase in E-cadherin (Fig. 6A). We observe that both AIB1 and MTA2 knockdowns resulted in significant changes in both EMT-cellular phenotype and motility competence (Fig. 6B, C, and D).

In order to gain further support for the role of the AIB1/MTA2 complex in endocrine resistant progression and regulation of cell adhesion/cadherin binding function, we again analyzed the ER^+^ endocrine resistant metastatic PDX models that had differential response to AIB1 inhibition and contrasting expression of CDH1 protein E-Cadherin (Fig. 6E). In E-Cadherin negative T638 (E-Cad^low^) AIB1, pAIB1 and MTA2 expression was significantly higher compared to E-Cadherin (E-Cad^high^) expressing T347 model (AIB1, *P* = 0.021; pAIB1, *P* = 0.001 and MTA2, *P* = 0.002) (Fig. 6F). E-Cadherin expression inversely correlated with AIB1 (*r* = −0.9305, *P* = 0.007), pAIB1 (*r* = −0.9130, *P* = 0.011) and MTA2 (*r* = −0.8684, *P* = 0.025) (Fig. 6G). Interestingly, forced overexpression of AIB1 in the primary cell culture derived from the T347 PDX (T347x) was sufficient to cause a reduction in E-Cadherin protein expression (Fig. 6H). Altogether, our data suggests that in endocrine resistant tumors, under estrogen deprivation, AIB1 partners with pro-metastatic proteins to drive disease progression through a uniquely acquired AIB1-dependent transcriptome and interactome enriched for adhesion and cadherin pathways.

## Discussion

In addition to the selection of the critical resistance mutations in PI3K/mTOR/AKT, ERBB2, cell cycle and ESR1 pathways described clinically, adaptations to endocrine therapy may also involve transcriptional and epigenomic reprogramming of key survival and pro-metastatic pathways which are triggered as cells acquire resistance. Here, we report that AIB1, in combination with MTA2, negatively regulates the expression of key nodes of cell adhesion/cadherin binding function. Consequently, this AIB1 mediated adaptation fine-tunes the regulation of migratory/growth potential of endocrine resistant cancer cells and activates a pro-metastatic phenotype. Our data provides evidence that AIB1 acts a survival factor in multiple endocrine resistant metastatic breast cancer models where AIB1 may also be a potential drug target [17, 36, 37]. We demonstrate successful targeting of endocrine resistant metastatic cell line and patient-derived tumors harboring high AIB1 and pAIB1 expression while presenting evidence of re-sensitization of resistant cells. Indeed, Gates et al. have previously reported pharmacological inhibition of AIB1 with a small molecule inhibitor SI-2 alone may be enough to re-sensitize resistant tumor to endocrine treatment in ESR1 mutant tumors in vivo [18].

Accumulating evidence indicates that aberrant AIB1 expression is a key event in multiple tumor types and is a regulator of tumor cell growth, survival, disease progression, and metastasis [6, 38]. Functionally, AIB1 executes its role in breast cancer by transcriptionally coactivating ER, even in the cases of treatment resistant mutant ER tumors [18, 39]. However, unbiased interrogation of the AIB1 interactome described here, revealed a new role for AIB1 as a facilitator of target gene repression in advanced endocrine resistance. Similar roles for steroid receptor coregulators have been described by our group and others [19, 28–32, 40]. Collectively these data provide evidence for a previously under-appreciated function of the SRC coregulator family in transcriptional suppression, broadening their impact and extending their regulatory network to promote metastasis.

Data presented in this study suggests that AIB1 contributes to metastatic disease progression, at least in part, independently of ER. This is pertinent considering the reported ER-independent mechanism of therapy resistance and the loss of ER expression reported in patient-matched ER^+^ primary and metastatic tumor samples [2, 41, 42]. It has been previously reported that AIB1 may function in the absence of ER to promote tumorigenesis [43] and such adaptations can result in the evolution of estrogen independence and estrogen deprivation treatment resistance [2]. It is less clear whether these changes are associated with resistance to selective ER degraders given that our experimental system models estrogen deprivation, achieved clinically via AI therapy. The ability of ER degraders to activate similar AIB1-driven pro-metastatic re-programming through these mechanisms may however be likely given the discordance between ER and AIB1 functions in metastatic tumors reported here.

This work describes a set of 114 AIB1 interacting proteins unique to the AI resistant phenotype. AIB1 directly interacts and colocalizes with MTA2 to repress *E-cadherin* expression thereby promoting EMT, migration and pro-metastatic phenotype of estrogen deprived breast cancer cells. Induction of EMT in concert with loss or dysfunction of *CDH1* is strongly linked with tumorigenesis and metastatic competence in numerous cancers [24]. Interestingly, AIB1 expression has previously correlated negatively with *E-cadherin* in pancreatic cancer, lung and MMTV-PyMt breast cancer mouse models [8, 36, 44]. Negative correlation between AIB1 and E-Cadherin will need to be confirmed in larger proteomic datasets considering both ductal and lobular breast cancers. However, here we provide a novel molecular mechanistic link between AIB1 and *CDH1* relevant to the pro-metastatic phenotype in endocrine resistance.

MTA2 is a member of the Mi-2/nucleosome remodeling and deacetylase (NuRD) complex which was previously shown to be important in mediating migration and anchorage-independent growth [34, 45]. Estrogen dependent interaction between AIB1 and MTA2 have previously been identified in screening studies in endocrine sensitive breast cancer cells [23], although the mechanism of action in the context of endocrine resistant tumors has not been described. In the resistant phenotype, it is feasible that in addition to the MTA2 having its own set of pre-marked DNA targets, AIB1 differentially recruits MTA2 to a specific set of targets pertinent to endocrine resistant cells. This raises the possibility that altered AIB1 interactome activity may result in coordinated resistant cell re-programming to promote the metastatic phenotype.

Although, we have focused on AIB1/MTA2 activity in the context of *CDH1* repression, it is likely that AIB1 recruits other transcriptional coregulators to carry out its pro-EMT function, indeed several known chromatin remodelers were also detected to directly interact with AIB1. In addition, although our investigation centers on the cell adhesion and cadherin pathways as the most enriched in resistant cells, components of multiple biological signaling pathways are enriched in the resistant cell specific AIB1 interactome. As such, AIB1 may coordinate multiple signaling pathways and biological processes that can contribute to alternative functions that support metastatic growth and which should be addressed in the future.

Our study is not without limitations. The precise dynamics of the AIB1 chromatin colocalization with MTA2 has yet to be explored. Here, through ChIP-re-ChIP experiments we have demonstrated colocalization of AIB1 and MTA2 specifically on the CDH1 promoter and presented evidence of dependence of MTA2 binding on AIB1. Whether AIB1 interacts with the entire protein complex simultaneously or if it sequentially recruits various coregulators to modulate transcription as described previously requires further investigation beyond the scope of this study [33]. Furthermore, it remains to be determined whether AIB1 recruitment alone, while necessary, is sufficient for the repressive function demonstrated here.

Data reported here present a mechanistic link between the aberrant expression of AIB1, its interactome and their contribution to breast cancer progression in endocrine resistance. Overall, these results provide new mechanistic insights into the potential targeting of AIB1 in the advanced setting.

## Materials and methods

### Bioinformatic analysis

Gene expression datasets on endocrine treated breast tumors were downloaded from NCBI GEO and previously published RNA-sequencing studies [15, 41, 46]. cBioPortal [47] was utilized to download metastatic breast cancer mutational and the Metabric data. Cistrome data browser (Cistrome DB) [27] was utilized to access ChIP-seq data [16]. Full details on all the analysis is provided in the Supplementary Methods File.

### Cell culture and functional assays

MCF7 cells were sourced from ATCC. Culture conditions and establishment protocols for the various endocrine resistant models (LetR [2], T347x [19], LTED [48], LCC9, and LY2 [20]) have been previously described. LetR cells were established from MCF7 cells stably overexpressing CYP19 and long-term treated in the presence of letrozole. All functional assays are described in the Supplementary Methods File.

### Animal studies

Full details on establishment of in vivo models is provided in the Supplementary Methods File.

Animal studies were performed in accordance with all the relevant ethical animal research regulations and were approved by Research Ethics Committee (#1045bbb) under a license from the HPRA. PDX Written and informed consent was acquired prior to collection of all patient tumor tissue under RCSI Institutional Review Board approved protocol (#13/09;ICORG09/07). PDX samples were established as described previously [49].

### Patient-derived tumor explants (PDTEs)

Metastatic PDX tissue was cut and dissected into 2-4mm^3^ tumor fragments and placed on 1 cm^3^ hemostatic gelatin dental sponges (Vetspon, Novartis) pre-soaked with human mammary epithelial media as described previously [50]. Full details provided in Supplementary Methods File.

### RNA-sequencing

RNA-seq was carried out in biological triplicate in MCF7 and LetR cells comparing siNT versus siAIB1. RNA was subjected to 100bpPE sequencing using the BGISEQ 500 platform.

### Protein profiling

Full details on protein extraction, immunoprecipitation, western blotting, and immunohistochemistry are provided in the Supplementary Methods File.

### RIME

RIME protocol was utilized to purify endogenous AIB1 and identify interacting proteins. Briefly steroid depleted cells were cross-linked, chromatin extracted and immunoprecipitated with either AIB1 antibody (10 µg; Santa Cruz; sc-25742) or negative control IgG (10 µg; Diagenode; C15410206) as previously described [22]. Full details are provided in the Supplementary Methods File.

### Chromatin immunoprecipitation (ChIP)

Steroid starved cells were fixed, crosslinked and ChIP was carried out as described in Supplementary Methods. ChIP-re-ChIP includes an additional elution step after first IP pull followed by the sequential ChIP pull with either antibody of interest or IgG [19].

### Statistical analysis

Wilcoxon-signed ranked tests were used with log2normCPM values in single gene expression queries. Differences between two groups were analyzed by two-tailed *t* test (Gaussian) or Mann–Whitney U-test (nonparametric) and were considered statistically different if *P* < 0.05. Survival data was calculated with the log-rank test. No statistical method was used to pre-determine sample size.

## Supplementary information

Supplementary Methods

Supplementary Figures

Supplementary Tables

## Data Availability

All data is included in the article and in its Supplementary Files. Sequencing data is deposited in the Gene Expression Omnibus under the accession number GSE158095.
