# Supplementary material for: Comparative analysis of the AIB1 interactome in breast cancer reveals MTA2 as a repressive partner which silences E-Cadherin to promote EMT and associates with a pro-metastatic phenotype
Source: Oncogene. 2021 Jan 8;40(7):1318–31. doi: 10.1038/s41388-020-01606-3 (PMC7892341; doi:10.1038/s41388-020-01606-3)
Supplement: Supplementary file 1 — Supplementary Methods [file 41388_2020_1606_MOESM1_ESM.pdf]

Comparative Analysis of the AIB1 interactome reveals MTA2 as a repressive partner silencing E-Cadherin to promote EMT and drive a pro-metastatic phenotype in endocrine resistant breast cancer

Damir Varešlija[1*], Elspeth Ward[1], Siobhan P. Purcell[1], Nicola S. Cosgrove[1], Sinéad Cocchiglia[1], Philip J. O'Halloran[2], Sara Charmsaz[1], Fiona T. Bane[1], Francesca M. Brett[3], Michael Farrell[3], Jane Cryan[3], Alan Beausang[3], Lance Hudson[1], Arran K. Turnbul[4], J. Michael Dixon[4], Arnold D.K. Hill[1], Nolan Priedigkeit[5], Steffi Oesterreich[5B], Adrian V. Lee[5A,B], Andrew H. Sims[6], Aisling M. Redmond[7], Jason S. Carroll[7], Leonie S. Young[1*]

Supplementary Methods

Materials and Methods

Bioinformatic analysis

Gene expression Survival analysis. Previously published microarray datasets were downloaded from NCBI GEO and these include AI- treated primary breast cancer datasets (GSE55374, GSE20181, and GSE41994), tamoxifen treated datasets (GSE66532, GSE9195, GSE17705 and GSE12093) and AI-treated metastatic ER^+ breast cancer (GSE41994). In analysis containing multiple array platforms, data was summarized with Ensemble alternative CDF and normalized with Robust Multi-array Average (RMA), before integration using ComBat to remove dataset-specific bias. *survivAll* R package was used to generate visual and numerical comparisons of all possible points of separation, enabling quantitative expression data to be reliably evaluated (1).

RNA-sequencing data on patient-matched primary breast cancer and metastasis (2-4) was obtained and raw CPM counts for genes of interest were analysed for differential expression. cBioPortal (5) was utilized to obtain mutational data with respect to metastatic breast cancer cohort and the Metabric cohort. Oncoplot and tables were generated using the built-in features and standard parameters. Oncoplot and tables were generated using the built-in features and standard parameters. cBioPortal was utilized for overall survival analysis of AIB1 mRNA expression. Default settings were used with automatically generated survival probabilities. Patients with deaths due to other or unknown causes are censored at the date of death and all other patients are censored at the date of last contact.

Cistrome data browser (Cistrome DB) (6) was utilized to access ChIP-seq data (7) and visualise the relevant binding peaks using the genome browser feature. Cistrome data browser (Cistrome DB) (6) was utilized to access ChIP-seq data (7) and visualise the relevant binding peaks using the genome browser feature. **Differential binding analysis of ChIP-Seq data**

Publicly available sequence alignment files from ChIP-Seq of AIB1,MTA2 and input control samples from MCF7 cell line were downloaded from the ENCODE portal (8) with the following identifiers: ENCFF722BQX, ENCFF976AUQ, ENCFF828SPJ, ENCFF043VGV, ENCFF583FAG, ENCFF049MPQ). Corresponding binding peak calls were downloaded from Cistrome DB (accession numbers: 101717, 101716, 63873, 63874). The DiffBind R package was used to identify a consensus peak set between AIB1 and MTA2 peak calls, differential binding analysis and visualization (9). The number of sequencing reads per ChIP sample overlapping each consensus peak were counted, and adjusted for the number of reads from input control sample. Differentially bound peaks were identified using DESeq2 and called statistically significant based on a false discovery rate (FDR) < 0.05. ChIPseeker R package was used to annotate both the consensus and differentially bound peak sets based on the hg19 reference human genome (10). ChIPseeker was further used to a) calculate and plot the percentage of peaks distributed in each gene feature and b) calculate and plot the distance of each peak relative to the transcription start site of the primary transcript for each gene.

Animal studies

Animal studies were performed in accordance with all the relevant ethical animal research regulations and were reviewed and approved by Research Ethics Committee (approval number 1045bbb) under a license from the HPRA. **Cell line derived xenografts.** Metastatic variants of the LY2 GFP-tagged cell line (LY2-LUC) (11) including $LY2^{CHEST\_WALL\_METS}$, $LY2^{LIVER\_METS}$, $LY2^{LUNG\_METS}$ and $LY2^{BONE\_METS}$ were established through *in vivo* selection. Briefly, 50000 cells were introduced via intracardiac injection into the left ventricle of 8 week old NOD-SCID mice. At the end of the *in vivo* experiment the presence of metastatic cells was confirmed by IVIS bioluminescence imaging (BLI) *in vivo* and *ex vivo.* Metastatic colonising cells from chest-wall, liver, lung and bone were isolated, digested and trypsinised, briefly

cultured and passaged through the mice via intracardiac injection three more times. Metastatic variants were established using same culture conditions as LY2 cell line model. All experiments were performed with early passage cells. **Patient derived xenografts (PDX).** Written and informed consent was acquired prior to collection of all patient tumor tissue under RCSI Institutional Review Board approved protocol (#13/09; ICORG09/07). PDX samples were established by processing and implanting tumor fragments into the mammary fat pad of 6 week-old NOD-SCID mice supplemented with 17β-estradiol pellets implanted sub-cutaneously as described (12). Upon successful engraftment, the PDX model was propagated by serial transplantation and biobanked. PDX tumors T638 and T347 have been previously validated and described (13). T328 is an ER+ve endocrine resistant tumor. All samples were sampled for IDEXX testing and histological studies. HCI-05 and -11 PDX models have been previously described (14).

RIME

The rapid immunoprecipitation mass spectrometry of endogenous proteins (RIME) was utilized to purify endogenous AIB1 and identify interacting proteins. After a 72 hr steroid depletion, the cells were seeded at 70% confluency for 24 hr into steroid depleted media with no hormone stimulation. At this point the cells were cross-linked, chromatin extracted and immunoprecipitated with either AIB1 antibody (10 µg; Santa Cruz; sc-25742) or negative control IgG (10 µg; Diagenode; C15410206). Raw data was processed, analyzed and results visualized as previously described (15). Those GO terms that were called as statistically significant from enrichment testing against the GO Molecular Function corpus were selected for plotting the msarc plot using the msarc package (version 1.4.5) in R based on the Mohammed *et al* methodology for visualisation (15).

Uniprot identifications (IDs) from MS experiments were mapped to Entrez IDs and gene symbols using the org.Hs.eg UNIPROT and org.Hs.eg SYMBOL tables from the org.Hs.eg.db package from Bioconductor version 3.4.1. The clusterProfiler package in R version 3.4.4 was used to functionally annotate proteins according to Gene Ontology molecular function (MF) categories using gene symbol ids as input (16). The hypergeometric test was used with significant GO terms called based on a bonferroni adjusted p-value<0.05 and a q-value<0.01 to control for the false discovery rate. Significant GO terms were visualised using the dotplot function.

Chromatin Immunoprecipitation (ChIP)

Steroid starved cells were fixed, crosslinked and ChIP was carried out as described previously (17). Chromatin was sonicated using Bioruptor Pico (Diagenode) and chromatin was subjected to immunoprecipitation with the AIB1 antibody (10 µg; Santa Cruz; sc-25742), MTA2 (10 µg; Santa Cruz; sc-55566) or negative control IgG (10 µg; Diagenode; Rabbit C15410206) bound to protein A Dynabeads (ThermoScientific). The proteins were then removed from the DNA by reverse crosslinking overnight and DNA was extracted using phenol-chloroform-isoamyl alcohol. q-PCR was carried out on eluted ChIP DNA utilising SYBR Green PCR (Qiagen). Primers sequences were: CDH1, F: CGTCTATGCGAGGCCGGGT; R: AACTGACTTCCGCAAGCTCACA. The delta delta (ΔΔ) Ct comparative method was used for analysis and data are presented as relative fold enrichment over IgG control. **ChIP-re-ChIP** was performed as described above with the addition of elution step after first IP pull followed by the sequential ChIP pull with either antibody of interest or IgG (13).

Cell culture and Functional assays

MCF7 cells were obtained from ATCC and cultured as recommended. Culture conditions and establishment protocols for the various endocrine resistant models (LetR (17), T347x (13),

LTED(18), LCC9, and LY2(19)) have been previously described. LetR cells were established from MCF7 cells stably overexpressing CYP19 and long-term treated in the presence of letrozole. For steroid depleted (SD) conditions, cells were washed with PBS and kept in PRF-MEM with 10% CDS-FBS, 1% l-glutamine for 96 hours and media changed every day. Cells used were authenticated (SourceBioScience) and regularly tested for mycoplasma contamination (LT07-118, Lonza). siRNAs directed against AIB1 (siAIB1-1: Dharmacon; L-003759-00-0005/ siAIB1-2: Thermo Fisher Scientific; s15700) and MTA2 (siMTA2-1: Dharmacon;L-008482-00/ siMTA2-2: Thermo Fisher Scientific; s17631) or relevant control scramble (NT siRNA) were used to knock down gene expression. Transfections were carried out using Lipofectamine 2000 (Invitrogen) as per manufacturer's instructions. The pcDNA3.1 plasmid containing full-length AIB1 was used for overexpression studies and empty pcDNA3.1 was used as a control plasmid. SI-2 (SML1762, Sigma), new class of potent small-molecule inhibitors for AIB1, has been described previously (20). Fulvestrant (ICI-182780) and Palbociclib (PD-0332991) were sourced from Selleckchem. **Mammopshere assay.** Cells were seeded in a 6 well ultra-low adherence plate (Corning, MA) (500 cells/well) and cultured in mammosphere media (DMEM F12 supplemented with EGF and B27 [Sigma]). Mammospheres were maintained and analysed as previously described (21). **EMT phenotype profiling.** LetR cells were steroid depleted for 72 hours and serum starved for 24 hours. The cells were plated into a 6-well plate and stimulated with 10% serum in the presence or absence of AIB1/MTA2. After 48 hours the cells were imaged and whole cell lysate was isolated for EMT protein profiling. **Migration and cell growth assays.** Cellomics Cell Motility Kit (Thermo Scientific) was used to measure individual cell migration after 24 hours as per manufacturer's instructions as previously described (17). Steroid starved and siRNA transfected cells were grown in 12-well plates (2000 cells/well) and manually counted using a haemocytometer.

Patient-derived tumor explants (PDTEs)

Metastatic PDX tissue was cut and dissected into 2-4mm^3 tumor fragments and placed on 1cm^3 hemostatic gelatin dental sponges (Vetspon, Novartis) pre-soaked with human mammary epithelial media as described previously (22). The *ex vivo* patient-derived tumor explants (PDTEs) were cultured and tumor pieces randomized to treatment with either SI-2 (50nM), 4-OHT (10^{-6}M) or DMSO for 72 hours after which they were paraffin embedded and IHC stained. IHC protein positivity levels of pAIB1, AIB1 and pER were quantified utilizing Aperio ImageScope software using the positive pixel algorithm. The investigator was not blinded during image analysis. The viability of the tumors was evaluated by utilizing ki67 as a proliferation marker to confirm viable, proliferating cells (22).

RNA-sequencing

RNA-seq was carried out in biological triplicate in MCF7 and LetR cells comparing siNT (Scramble siRNA) cells versus cells depleted of AIB1 using a SMARTpool ON-TARGETplus siRNA. RNA was extracted using Qiagen RNeasy Kit and subjected to 100bp PE sequencing using the BGISEQ 500 platform. Briefly, clean reads were mapped to reference genome using Bowtie (23) and gene expression was calculated with RSEM (24). Differentially expressed genes (DEGs) were detected with DEseq2 (25). With the KEGG pathway analysis, the pathway functional enrichment was performed using phyper, a function of R.

Protein studies

Cell nuclear fraction was lysed using NE-PER kit (Thermo-Fisher Scientific). EMT profiling experiments were carried out with whole cell lysates. In the case of PDX tumors, biological replicates were mixed, minced and lysed in ice cold RIPA buffer while sonicating with a Bioruptor Pico (Diagenode). For co-immunoprecipitation experiments, 400 µg of nuclear protein was immunoprecipitated (IP) with AIB1 (10 µg, SC-25742) or IgG (10 µg, C15410206)

using Dynabeads Protein A (10002D, Life Technologies) following standard IP protocol. Protein extracted for western blots or IPs was electrophoresed and immunoblotted with AIB1 (1/200; Santa Cruz; sc-25742), MTA2 (1/250, Santa Cruz; sc-55566), E-Cadherin (1/250; Novacastra Leica; NCL-L-E-CAD), phosphorylated-AIB1 (Thr24) (1:1000; 2979S, Cell signaling), phosphorylated-AIB1 (Ser857) (1/1000, Thermo Fisher Scientific, PA5-64836), Vimentin (D21H3, Cell Signaling), N-Cadherin (D4R1H, Cell Signaling), Snail (C15D3, Cell Signaling), Slug (C19G7, Cell Signaling), TBP (1/1000; Cell Signalling; 8515) and B-Actin (1/4000; Abcam; ab6276). **Immunohistochemistry (IHC)**

IHC was performed with antibodies against AIB1 (1:200, rabbit, sc-25742, Santa Cruz), KI67 (1:100, mouse monoclonal, Dako, MIB1, M2740), phosphorylated-AIB1 (Thr24) (2979S, Cell signaling) phosphorylated-AIB1 (Ser857) (1/1000, Thermo Fisher Scientific, PA5-64836), phosphorylated ER SER118 (2511S, Cell Signaling) and MTA2 (Sigma) using Dako EnVision^{TM} Kit (17). Antigen retrieval and primary antibody incubations were carried out as per manufacturer’s instructions. Positivity scores were assessed and scored utilizing a modified Allred IHC scoring system and the H-score for nuclear protein expression.

References

1. Pearce DA, Nirmal AJ, Freeman TC, Sims AH. Continuous Biomarker Assessment by Exhaustive Survival Analysis. bioRxiv. 2018.
2. Priedigkeit N, Watters RJ, Lucas PC, Basudan A, Bhargava R, Horne W, et al. Exome-capture RNA sequencing of decade-old breast cancers and matched decalcified bone metastases. JCI Insight. 2017;2(17).
3. Vareslija D, Priedigkeit N, Fagan A, Purcell S, Cosgrove N, O'Halloran PJ, et al. Transcriptome Characterization of Matched Primary Breast and Brain Metastatic Tumors to Detect Novel Actionable Targets. J Natl Cancer Inst. 2018.
4. Siegel MB, He X, Hoadley KA, Hoyle A, Pearce JB, Garrett AL, et al. Integrated RNA and DNA sequencing reveals early drivers of metastatic breast cancer. J Clin Invest. 2018;128(4):1371-83.
5. Cerami E, Gao J, Dogrusoz U, Gross BE, Sumer SO, Aksoy BA, et al. The cBio cancer genomics portal: an open platform for exploring multidimensional cancer genomics data. Cancer Discov. 2012;2(5):401-4.
6. Mei S, Qin Q, Wu Q, Sun H, Zheng R, Zang C, et al. Cistrome Data Browser: a data portal for ChIP-Seq and chromatin accessibility data in human and mouse. Nucleic Acids Res. 2017;45(D1):D658-D62.
7. Jansen MP, Knijnenburg T, Reijm EA, Simon I, Kerkhoven R, Droog M, et al. Hallmarks of aromatase inhibitor drug resistance revealed by epigenetic profiling in breast cancer. Cancer Res. 2013;73(22):6632-41.
8. Davis CA, Hitz BC, Sloan CA, Chan ET, Davidson JM, Gabdank I, et al. The Encyclopedia of DNA elements (ENCODE): data portal update. Nucleic Acids Res. 2018;46(D1):D794-D801.
9. Ross-Innes CS, Stark R, Teschendorff AE, Holmes KA, Ali HR, Dunning MJ, et al. Differential oestrogen receptor binding is associated with clinical outcome in breast cancer. Nature. 2012;481(7381):389-93.
10. Yu G, Wang LG, He QY. ChIPseeker: an R/Bioconductor package for ChIP peak annotation, comparison and visualization. Bioinformatics. 2015;31(14):2382-3.
11. McBryan J, Fagan A, McCartan D, Bane FT, Vareslija D, Cocchiglia S, et al. Transcriptomic Profiling of Sequential Tumors from Breast Cancer Patients Provides a Global View of Metastatic Expression Changes Following Endocrine Therapy. Clin Cancer Res. 2015;21(23):5371-9.
12. Vareslija D, Cocchiglia S, Byrne C, Young L. Patient-Derived Xenografts of Breast Cancer. Methods Mol Biol. 2017;1501:327-36.
13. Ward E, Vareslija D, Charmsaz S, Fagan A, Browne AL, Cosgrove NS, et al. Epigenome-wide SRC-1 mediated gene silencing represses cellular differentiation in advanced breast cancer. Clin Cancer Res. 2018.
14. DeRose YS, Wang G, Lin YC, Bernard PS, Buys SS, Ebbert MT, et al. Tumor grafts derived from women with breast cancer authentically reflect tumor pathology, growth, metastasis and disease outcomes. Nat Med. 2011;17(11):1514-20.
15. Mohammed H, D'Santos C, Serandour AA, Ali HR, Brown GD, Atkins A, et al. Endogenous purification reveals GREB1 as a key estrogen receptor regulatory factor. Cell Rep. 2013;3(2):342-9.
16. Yu G, Wang LG, Han Y, He QY. clusterProfiler: an R package for comparing biological themes among gene clusters. OMICS. 2012;16(5):284-7.
17. Vareslija D, McBryan J, Fagan A, Redmond AM, Hao Y, Sims AH, et al. Adaptation to AI Therapy in Breast Cancer Can Induce Dynamic Alterations in ER Activity Resulting in Estrogen-Independent Metastatic Tumors. Clin Cancer Res. 2016;22(11):2765-77.
18. Nguyen VT, Barozzi I, Faronato M, Lombardo Y, Steel JH, Patel N, et al. Differential epigenetic reprogramming in response to specific endocrine therapies promotes cholesterol biosynthesis and cellular invasion. Nat Commun. 2015;6:10044.

19. Browne AL, Charmsaz S, Vareslija D, Fagan A, Cosgrove N, Cocchiglia S, et al. Network analysis of SRC-1 reveals a novel transcription factor hub which regulates endocrine resistant breast cancer. Oncogene. 2018.
20. Rohira AD, Yan F, Wang L, Wang J, Zhou S, Lu A, et al. Targeting SRC Coactivators Blocks the Tumor-Initiating Capacity of Cancer Stem-like Cells. Cancer Res. 2017;77(16):4293-304.
21. Shaw FL, Harrison H, Spence K, Ablett MP, Simoes BM, Farnie G, et al. A detailed mammosphere assay protocol for the quantification of breast stem cell activity. J Mammary Gland Biol Neoplasia. 2012;17(2):111-7.
22. Shafi AA, Schiewer MJ, de Leeuw R, Dylgjeri E, McCue PA, Shah N, et al. Patient-derived Models Reveal Impact of the Tumor Microenvironment on Therapeutic Response. Eur Urol Oncol. 2018;1(4):325-37.
23. Langmead B, Salzberg SL. Fast gapped-read alignment with Bowtie 2. Nat Methods. 2012;9(4):357-9.
24. Li B, Dewey CN. RSEM: accurate transcript quantification from RNA-Seq data with or without a reference genome. BMC Bioinformatics. 2011;12:323.
25. Love MI, Huber W, Anders S. Moderated estimation of fold change and dispersion for RNA-seq data with DESeq2. Genome Biol. 2014;15(12):550.
