# Supplementary material for: Comparative analysis of the AIB1 interactome in breast cancer reveals MTA2 as a repressive partner which silences E-Cadherin to promote EMT and associates with a pro-metastatic phenotype
Source: Oncogene. 2021 Jan 8;40(7):1318–31. doi: 10.1038/s41388-020-01606-3 (PMC7892341; doi:10.1038/s41388-020-01606-3)
Supplement: Supplementary file 2 — Supplementary Figures [file 41388_2020_1606_MOESM2_ESM.pdf]

Comparative analysis of the AIB1 interactome in breast cancer reveals MTA2 as a repressive partner which silences E-Cadherin to promote EMT and associates with a pro-metastatic phenotype

Damir Varešlija, Elspeth Ward, Siobhan Purcell, Nicola Cosgrove, Sinéad Cocchiglia, Fiona T. Bane, Philip J. O'Halloran, Sara Charmsaz, Francesca M. Brett, Michael Farrell, Jane Cryan, Alan Beausang, Lance Hudson, Arran Turnbul, J. Michael Dixon, Arnold D.K. Hill, Nolan Priedigkeit, Steffi Oesterreich, Adrian V. Lee, Andrew H. Sims, Aisling M. Redmond, Jason S. Carroll, Leonie S. Young

Data Supplement

Supplementary Figure 1

A

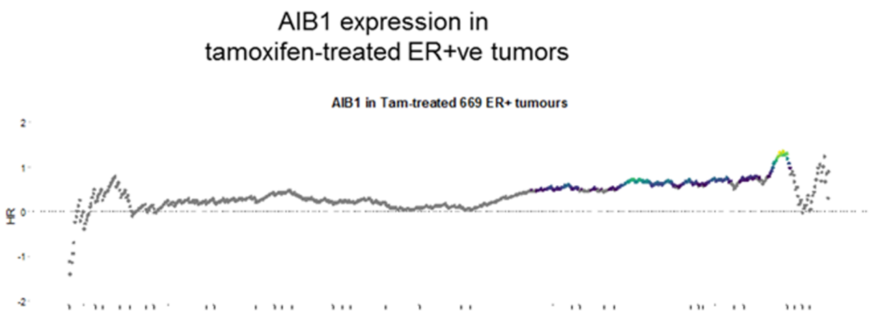

B

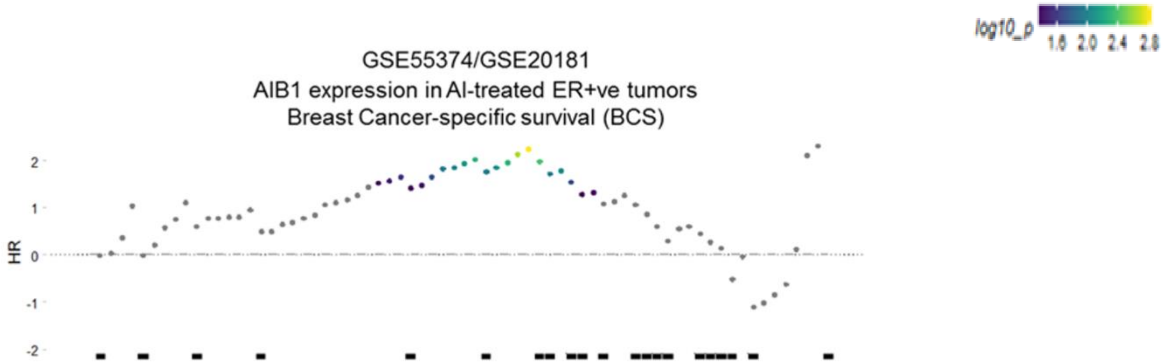

C

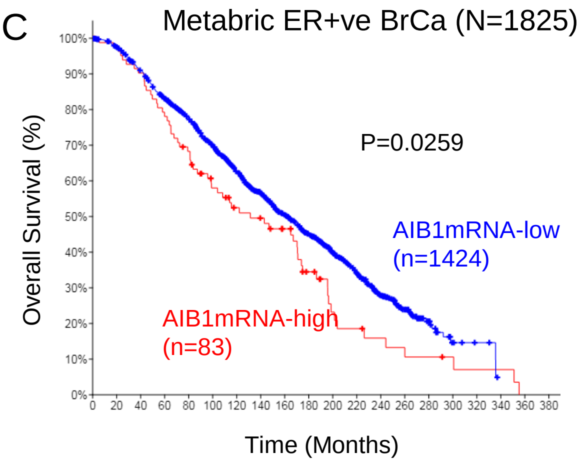

D

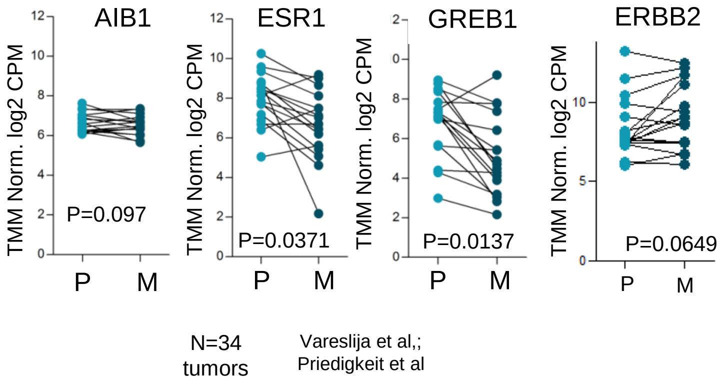

Number of Cases, Total	Number of Cases, Deceased	Median Months Overall	Median Months Overall
AIB1 mRNA-high	**83**	**62**	**131.33**
AIB1 mRNA-low	**1376**	**791**	**161.67**

E

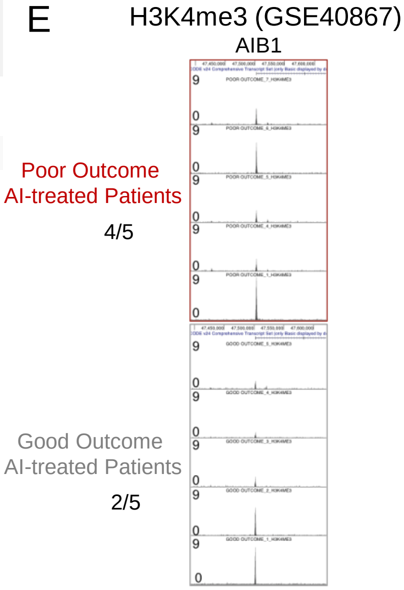

Figure S1 (Related to Figure 1). (A) Ranked AIB1 expression in primary breast tumors from ER positive AI-treated patients. AIB1 mRNA expression associated significantly with worse breast cancer specific survival (BCS) (p=0.0004, n=70, HR 4.7). Data is from published Affymetrix microarray datasets (GSE55374/GSE20181). **(B)** Ranked AIB1 target gene set expression in 669 primary breast tumors from ER positive 4-OHT-treated patients. Colors are log2 mean-centered values. Data is from four published Affymetrix microarray datasets (GSE6532, GSE9195, GSE17705, GSE12093). Kaplan–Meier analysis of distant metastatic free survival (DMFS) according to mRNA expression of AIB1 in ER positive 4-OHT-treated patients (n=669). **(C)** High AIB1 mRNA expression associated with worse overall survival (p=0.0259, n=1825). Data is from published METABRIC cohort and Kaplan-Meier was generated in cBioPortal. **(D)** Paired ladder plot of ESR1, GREB1 and AIB1 mRNA expression in patient-matched primary (P) and metastatic (M) ER+ cases (n=17 patients; n=34 tumors). Light green dots represent primary tumor expression values and dark green dots represent metastatic tumor expression values (log2norm CPM). P value obtained via two-sided Wilcoxon signed-rank test. **(E)** Genome browser snapshot illustrating AIB1 gene- and MTA2 gene-related binding events for H3K4me3 in poor outcome (red) and good outcome (grey) epigenomically profiled tumor samples (GSE40867). Binding intensity scale adjusted from 0-9. Peaks with a binding peak intensity value greater than 2.5 considered a positive binding event.

Supplementary Figure 2

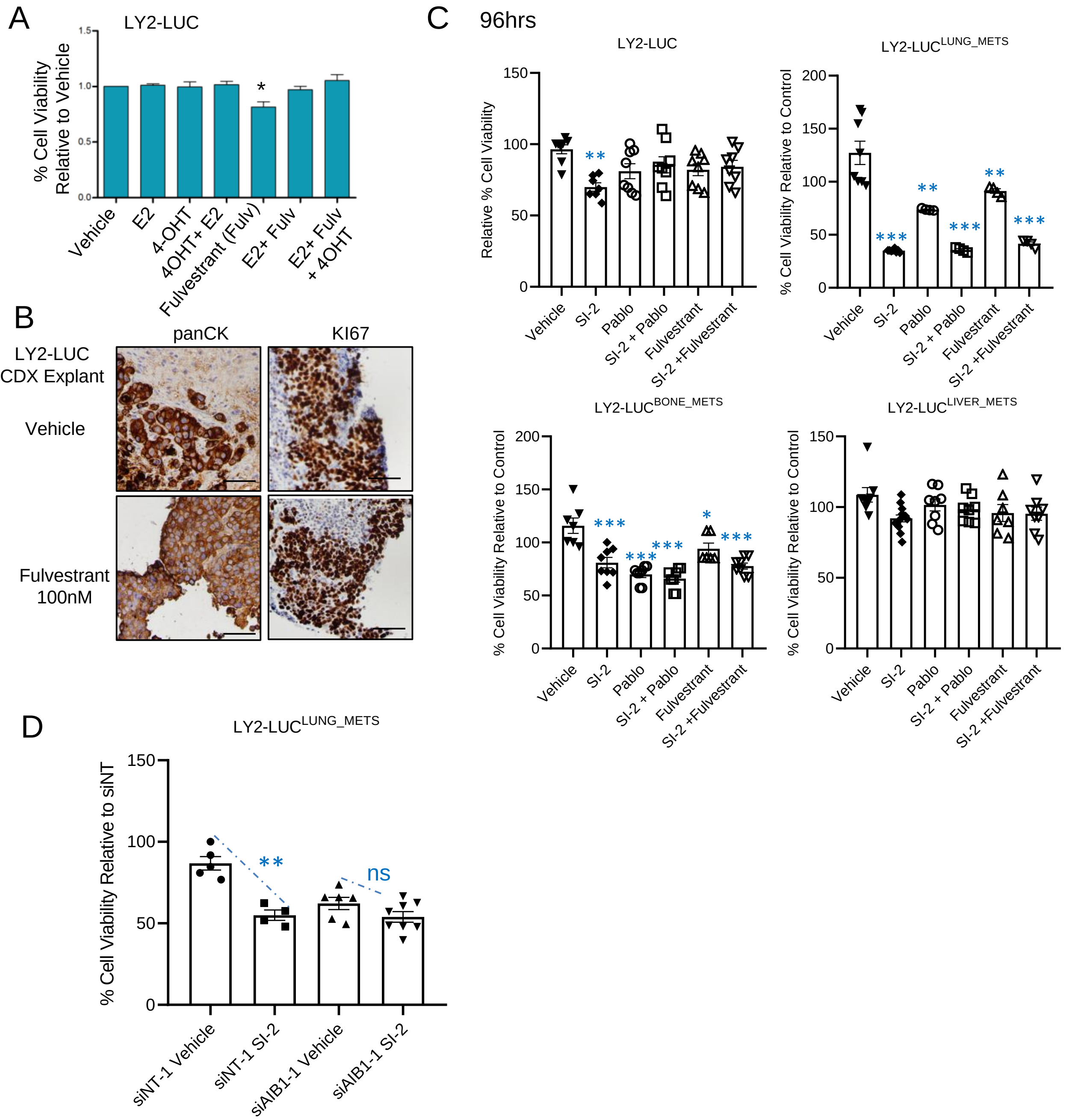

Figure S2 (Related to Figure 2). (A) LY2-luc parental cells were steroid depleted for 72 hr and treated for 96 hours with vehicle (DMSO) or various combinations of E2 (10^{-7}M), 4-OHT (10^{-7}M) and ICI Fulvestrant (10^{-7}M). (B) LY2-luc cells were injected into the mammary fat pad (10^{6}; 50:50 ration of PBS/Matrigel) and allowed to grow to 150mm^{3}). Tumors were resected and set up in duplicate for tumor explants as described in methods and Figure 3. The LY2-luc tumor explants were treated with either vehicle or 10^{-7}M ICI over 72 hours. Immunohistochemistry (IHC) protein analysis of panCK (20x) and ki67 (10x) on the treated tumors formalin-fixed and paraffin embedded. Image representative of n=2. At least 500 cells were assessed in each case. (C) Cells were treated for 96 hrs with vehicle (DMSO), SI-2 (50nM), fulvestrant (100nM) or palbociclib (1µM) under estrogen deprived conditions. (D) LY2-LUC^{LUNG_METs} cells were transfected with either siNT-1 or siAIB1-1. After 24 hours cells were seeded out and treated for 96 hours with vehicle (DMSO) or SI-2 (50nM). Cell viability was measured using an MTS assay and graphs display % cell viability relative to vehicle. Two-sided t-tests were used to calculate P values (***P<0.0001).

Supplementary Figure 3

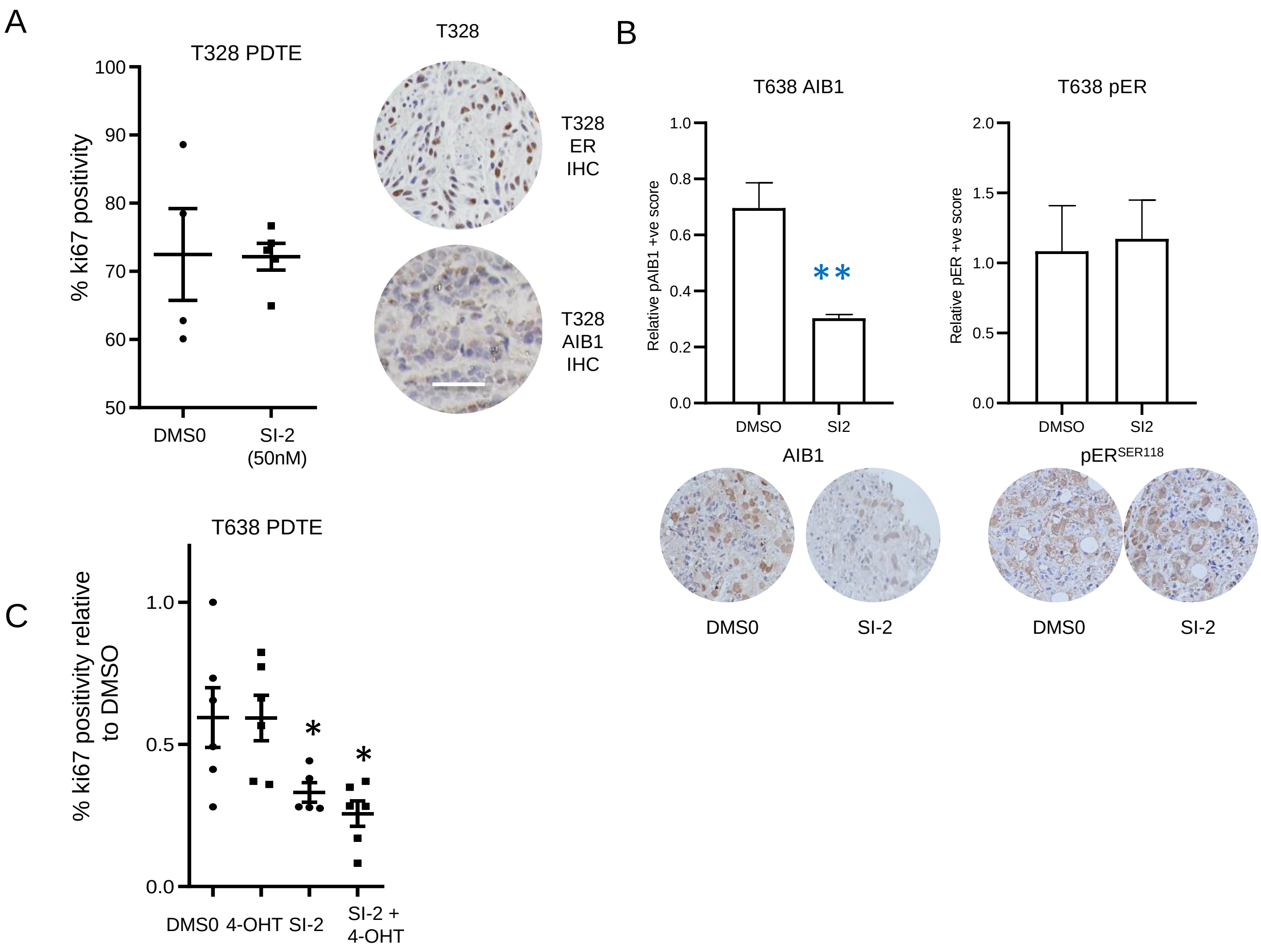

Figure S3 (Related to Figure 3). (A) ER+ve endocrine resistant PDTE, T328, was treated with with DMSO or 50nM SI-2 and processed as described in Figure 3.Bar chart displays ki67% determined from manual counts of ki67 positive cells over total number of cells. Representative images of IHC staining for ER and AIB1. (B) Bar chart displays relative AIB1 and pER^{SER118} IHC Scores determined by Aperio ImageScope positive pixel algorithm. % positivity is positive cells over total number of cells. Representative images of IHC staining for AIB1 and pER^{SER118}. (C) T638 PDTE was treated with DMSO, 50nM SI-2 or 10^{-6}M 4-OHT and processed as described. Bar chart displays ki67% determined from manual counts of ki67 positive cells over total number of cells. All scale bars represent 50 µm. Error bars represent mean ± s.e.m. (n = 4–6 images per group). Two-sided t-tests were used to calculate P values

Supplementary Figure 4

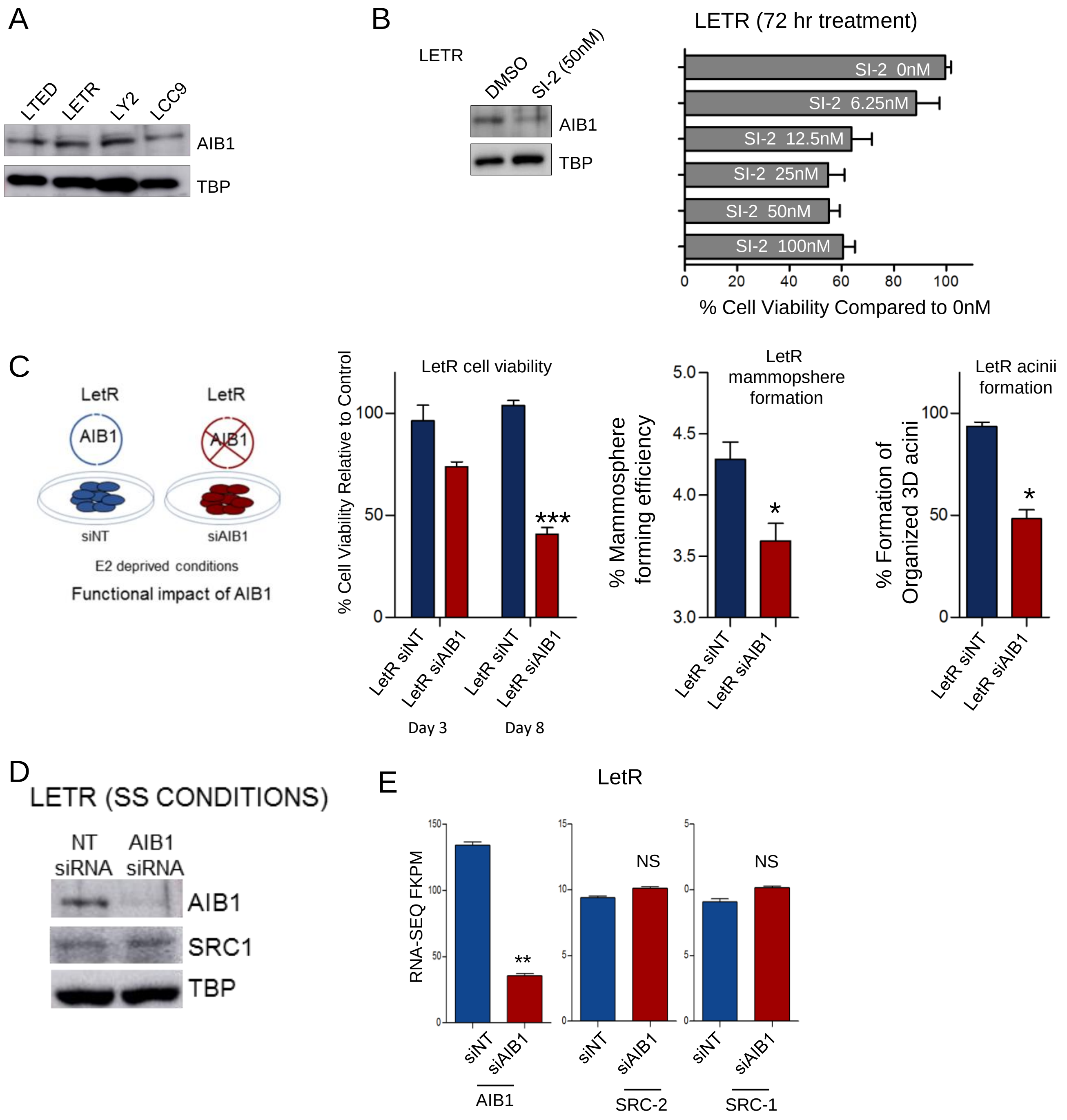

Figure S4 (Related to Figure 4). (A) Western blotting of AIB1 and TBP protein expression in the nuclear lysates of various endocrine resistant metastatic variants under estrogen deprived conditions. (B) AIB1 expression after 48 hr in SI-2 treated LetR cells. Representative western blot images of n=3. LetR cells treated with increasing concentration of SI-2. Cell viability measured after 72 hr using MTS assay. (C) AI resistant, LetR cells, were depleted of AIB1 using a SMARTpool ON-TARGETplus siRNA (10nM). All functional experiments were carried out under estrogen deprived conditions. (Cell viability assay) LetR cells were seeded at $2x10^4$ cells/well in 12-well plates. Growth assay was measured by manual cell counts at three different time points. Graph shows total cell number over an 8-day period (Day 3 and Day 8 measurements were taken). (Mammosphere assay) AIB1 siRNA LetR cells have less mammosphere forming efficiency (MFE) compared to control. (Acini Assay) Cells were assessed for acini formation in Matrigel for 21 days. LetR cells transfected with non-targeting Scramble siRNA (NT) fail to form acini with a significant degree of epithelial organisation. LetR cells that are transfected with siRNA against AIB1 are able to form better 3D organised structures with a hollow lumen. Chart displays the percentage of cells that formed acini with epithelial organisation. (Cell Motility) Cellomics Cell Motility Kit was used to assess individual cell movement in collagen in a 96-well plate after 24 hr. Representative images are shown as seen on the microscope after cells were fixed and stained (20X magnification, scale bars represent 20 µm). Image of treatment sensitive cell line MCF7 is shown for comparison. Histogram shows mean migratory area per cell (μm^2). All results are mean ± S.E.M., n=3 and two-sided t-tests were used to calculate P values. (D) Western blotting of AIB1 and SRC1 protein expression in nuclear extract of LetR cells. TBP used as a loading control. AI resistant, LetR cells, were depleted of AIB1 using a SMARTpool ON-TARGETplus siRNA (10 nM). All functional experiments were carried out under estrogen deprived conditions (SS) and cell viability, mammosphere and 3D acini assays were set up from the same transfection 6-well plate and considered valid only after confirmed protein knockdown. Same protein lysates were immunoblotted for SRC1 to demonstrate specificity of the siRNA knockdown. (E) FKMP values relating to the expression of SRC-1, SRC-2 and AIB1 in LetR NT and LetR AIB1 siRNA from RNA-seq data.

Supplementary Figure 5

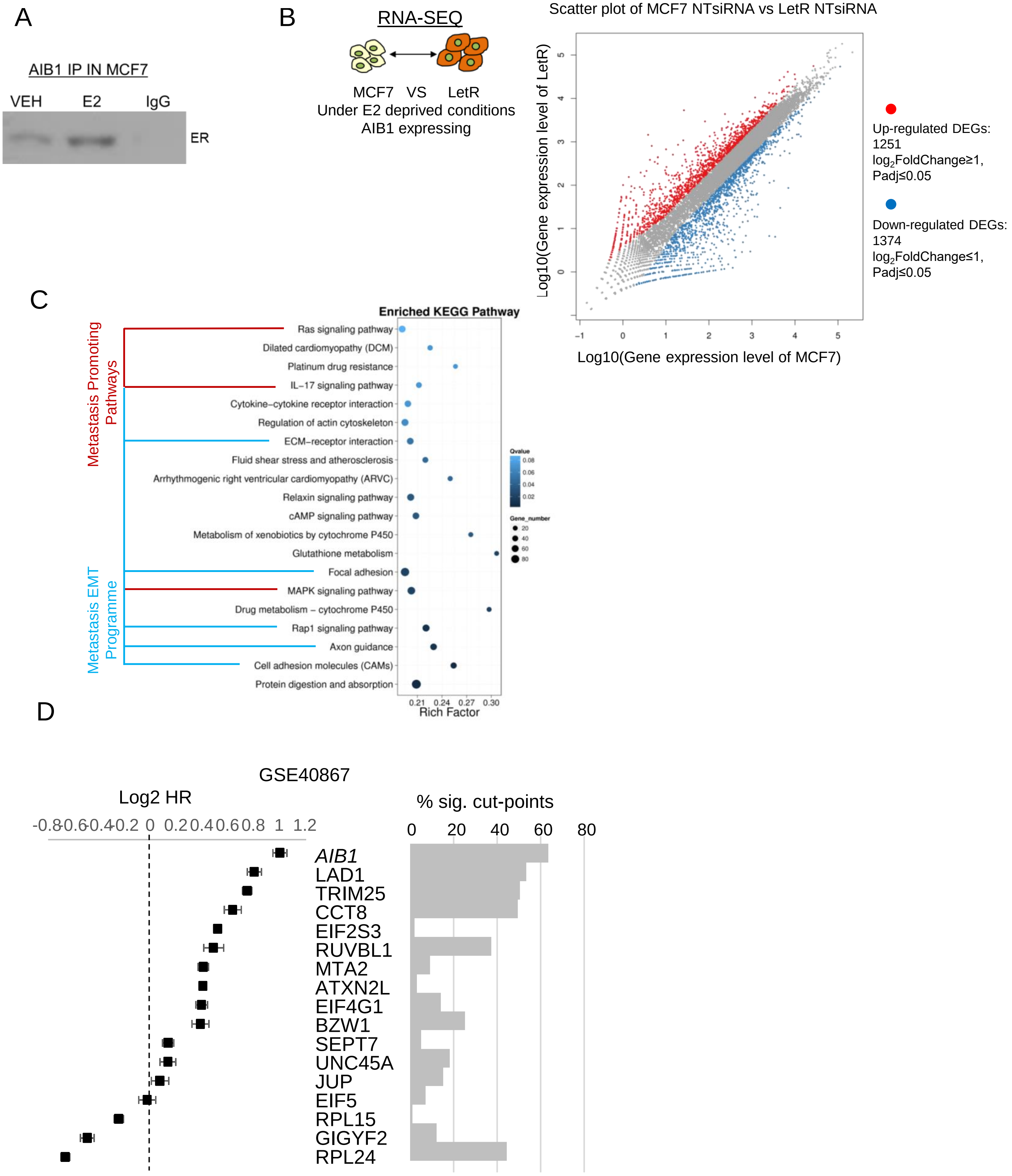

Figure S5. (A) Co-Immunoprecipitation of AIB1-ER followed by western immunoblotting to detect the ER interaction in nuclear extracts of steroid deprived LetR cells treated with either vehicle (0.001% Ethanol) or estrogen (E2; 10^{-7}M) for 50 mins. IgG antibody used as a control. (B) Schematic of the RNA-seq experimental design. RNA-seq comparing MCF7 vs LetR under estrogen deprived conditions without AIB1 knockdown (n=3 biological replicate). Scatter plot displays top differentially upregulated (n=1251) and downregulated (n=1374) genes identified in LetR compared to MCF7 cells (log2FoldChange≥1, Padj≤0.05). (C) Plot of the KEGG pathway enrichment test for the differentially expressed genes specific to LetR cells. X-axis represents enrichment factor. Y axis represents pathway name. The color indicates the q-value (high: white, low: blue), the lower q-value indicates the more significant enrichment. Point size indicates DEG number (The bigger dots refer to larger amount). Rich Factor refers to the value of enrichment factor, which is the quotient of foreground value (the number of DEGs) and background value (total Gene amount). (D) Comprehensive survival analysis (GSE40867, N=99) showing the average log2 hazard ratio (+/- standard error) and the proportion of significant ($p<0.05$) cut-points for the 16 top-ranked AIB-1 interacting proteins (AIB1 shown in italics for reference). White–gray–black bars indicate significance of all possible cut points from P = 1 to 0.001.

Supplementary Figure 6

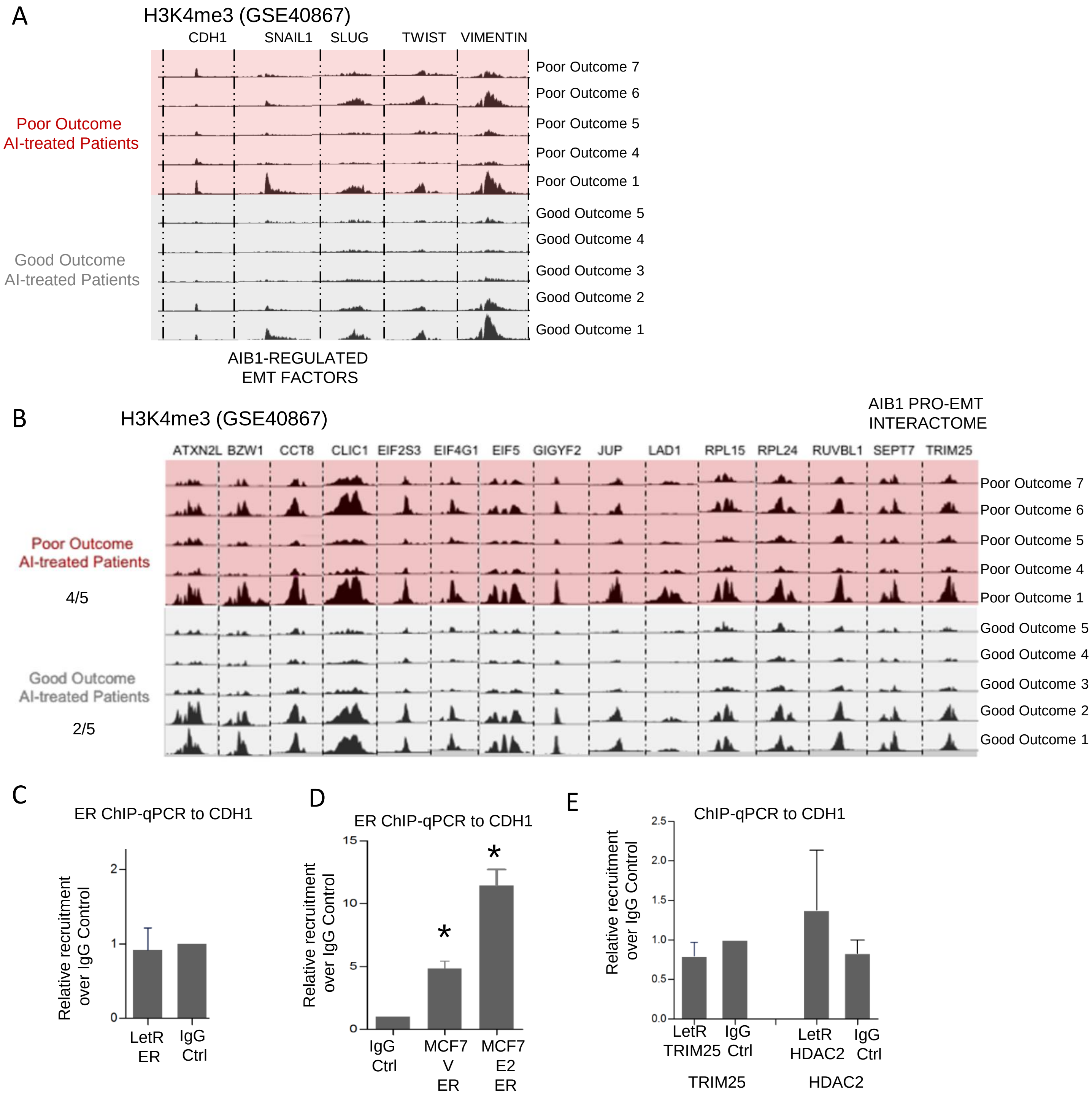

Figure S6 (Related to Figure 5). Genome browser snapshot illustrating binding events for H3K4me3 in poor outcome (red) and good outcome (grey) epigenomically profiled tumor samples (GSE40867). Target genes illustrated include those enriched for (A) AIB1-regulated EMT factors and (B) AIB1 RIME interacting proteins enriched for pathways of cell adhesion and cadherin binding. Binding intensity scale adjusted from 0-10 and standardized across all genes. (C) ER ChIP assay in LetR cells showing recruitment of ER to the CDH1 promoters in the absence of steroid treatment under estrogen deprived conditions. CHIP-grade IgG antibody was used as a control and data are presented as the relative enrichment over IgG. (D) ER ChIP assay in MCF7 cells showing recruitment of ER to the CDH1 promoters under either vehicle or estrogen-treated ($10\text{-}7^{M}$) condition. CHIP-grade IgG antibody was used as a control and data are presented as the relative enrichment over IgG. (E) TRIM25 and HDAC2 ChIP assay in LetR cells showing recruitment of ER to the CDH1 promoters in the absence of steroid treatment under estrogen deprived conditions. CHIP-grade antibody was used as a control and data are presented as the relative enrichment over IgG. (F) Following MTA2 siRNA transient knockdown, CDH1 mRNA was quantified and MTS assay was used to measure cell viability. Western blotting of MTA2 and B-actin protein expression in the lysates of LetR cells under estrogen deprived conditions is shown. (qPCR) Grey bar chart displays qRT-PCR analysis of CDH1 gene expression in the LetR cells transiently transfected with either NT or MTA2 siRNA. (Cell Viability Assay) LetR cells were seeded at $2x10^4$ cells/well in 12-well plates. Growth assay was measured by manual cell counts at three different time points. Graph shows total cell number over an 8-day period. In each case three biologically independent replicates were utilised and all statistical tests were carried out with two-sided t-tests.

Supplementary Figure 7

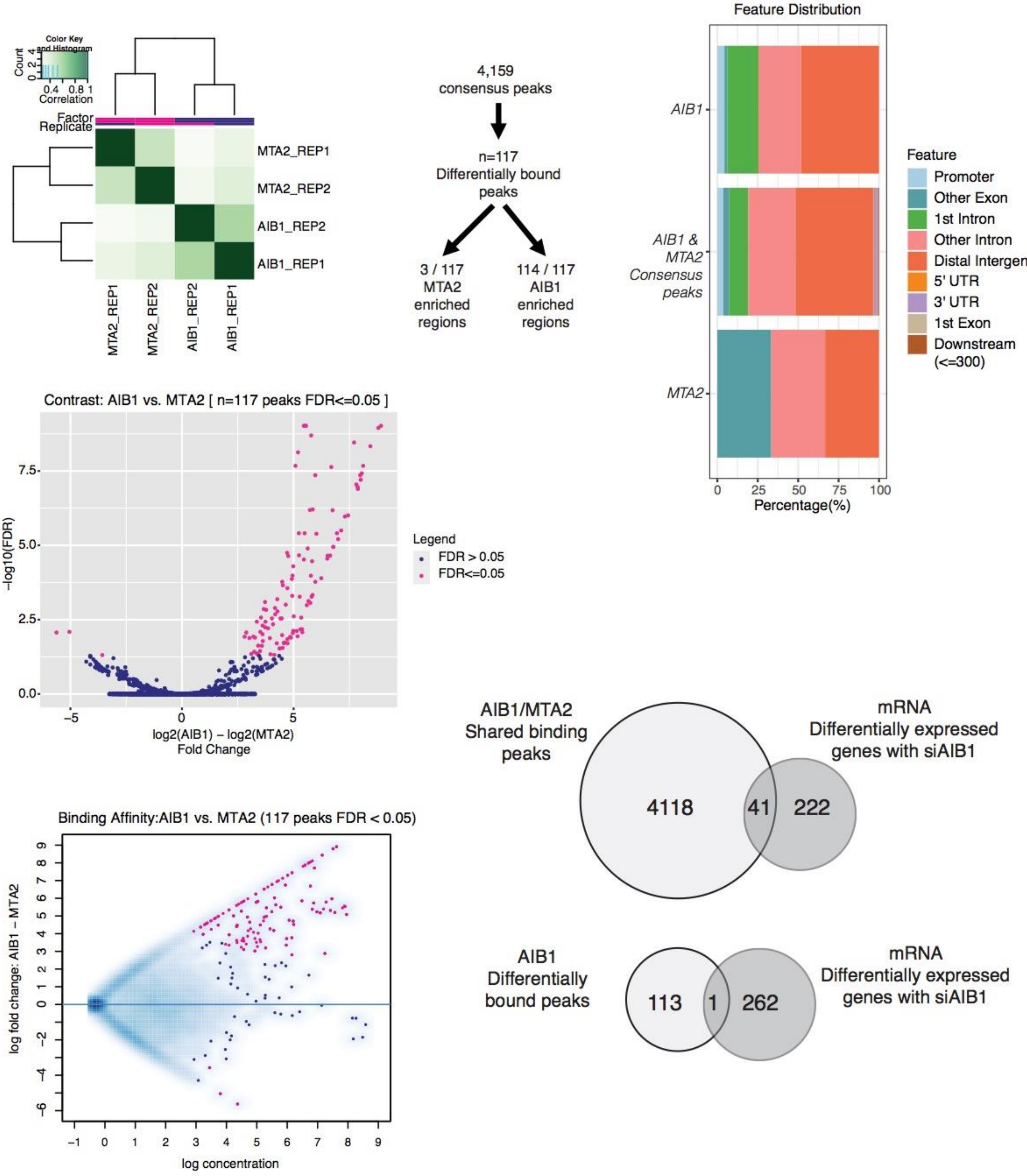

Figure S7 (Related to Figure 5). Consensus and differential binding analysis of AIB1 and MTA2 in endocrine sensitive cell line. A) Correlation heatmap representing unsupervised hierarchical clustering of ChIP samples in replicate. Sample-sample correlation scores calculated from normalised ChiP sequencing read counts. B) Summary diagram of the number of consensus peaks and differentially bound peaks detected from ChIP-Seq of AIB1 and MTA2 in replicate using DiffBind R package. C) Horizontal stacked barplot of the % distribution of annotated AIB1 enriched, AIB1 & MTA2 consensus peak set and MTA2 enriched peaks respectively across genomic features. Peaks were annotated based on the hg19 human reference genome using the ChIPseeker R package. D) Volcano plot of significantly differentially bound sites in AIB1 vs MTA2 ChIP samples. Log2 fold change values on the x axis and corresponding negative log scale 10 FDR confidence statistic from DESeq2 on y axis. Statistically significant differentially bound peaks are coloured in pink at a FDR < 0.05. E) Binding affinity MA plot: AIB1 vs MTA2. Data points in blue represent a consensus binding site, with differentially bound peaks highlighted in pink. Log2 fold change value (y axis) vs log concentration (log2 mean normalised number of reads across all samples for each consensus binding site) x axis. F) Venn diagrams representing the overlap of AIB1-MTA2 shared binding peakset (n = 4,159) and statistically significant differentially bound peaks (n = 117; FDR < 0.05) respectively with AIB1 siRNA differentially expressed genes (n= 263; FDR < 0.05).
